# Jet energy resolution in proton-proton collisions at $\sqrt{\mathrm{s}}=7\mbox{ TeV}$ recorded in 2010 with the ATLAS detector

**DOI:** 10.1140/epjc/s10052-013-2306-0

**Published:** 2013-03-02

**Authors:** The ATLAS Collaboration, G. Aad, T. Abajyan, B. Abbott, J. Abdallah, S. Abdel Khalek, A. A. Abdelalim, O. Abdinov, R. Aben, B. Abi, M. Abolins, O. S. AbouZeid, H. Abramowicz, H. Abreu, E. Acerbi, B. S. Acharya, L. Adamczyk, D. L. Adams, T. N. Addy, J. Adelman, S. Adomeit, P. Adragna, T. Adye, S. Aefsky, J. A. Aguilar-Saavedra, M. Agustoni, M. Aharrouche, S. P. Ahlen, F. Ahles, A. Ahmad, M. Ahsan, G. Aielli, T. Akdogan, T. P. A. Åkesson, G. Akimoto, A. V. Akimov, M. S. Alam, M. A. Alam, J. Albert, S. Albrand, M. Aleksa, I. N. Aleksandrov, F. Alessandria, C. Alexa, G. Alexander, G. Alexandre, T. Alexopoulos, M. Alhroob, M. Aliev, G. Alimonti, J. Alison, B. M. M. Allbrooke, P. P. Allport, S. E. Allwood-Spiers, J. Almond, A. Aloisio, R. Alon, A. Alonso, F. Alonso, B. Alvarez Gonzalez, M. G. Alviggi, K. Amako, C. Amelung, V. V. Ammosov, A. Amorim, N. Amram, C. Anastopoulos, L. S. Ancu, N. Andari, T. Andeen, C. F. Anders, G. Anders, K. J. Anderson, A. Andreazza, V. Andrei, X. S. Anduaga, P. Anger, A. Angerami, F. Anghinolfi, A. Anisenkov, N. Anjos, A. Annovi, A. Antonaki, M. Antonelli, A. Antonov, J. Antos, F. Anulli, M. Aoki, S. Aoun, L. Aperio Bella, R. Apolle, G. Arabidze, I. Aracena, Y. Arai, A. T. H. Arce, S. Arfaoui, J-F. Arguin, E. Arik, M. Arik, A. J. Armbruster, O. Arnaez, V. Arnal, C. Arnault, A. Artamonov, G. Artoni, D. Arutinov, S. Asai, R. Asfandiyarov, S. Ask, B. Åsman, L. Asquith, K. Assamagan, A. Astbury, M. Atkinson, B. Aubert, E. Auge, K. Augsten, M. Aurousseau, G. Avolio, R. Avramidou, D. Axen, G. Azuelos, Y. Azuma, M. A. Baak, G. Baccaglioni, C. Bacci, A. M. Bach, H. Bachacou, K. Bachas, M. Backes, M. Backhaus, E. Badescu, P. Bagnaia, S. Bahinipati, Y. Bai, D. C. Bailey, T. Bain, J. T. Baines, O. K. Baker, M. D. Baker, S. Baker, E. Banas, P. Banerjee, Sw. Banerjee, D. Banfi, A. Bangert, V. Bansal, H. S. Bansil, L. Barak, S. P. Baranov, A. Barbaro Galtieri, T. Barber, E. L. Barberio, D. Barberis, M. Barbero, D. Y. Bardin, T. Barillari, M. Barisonzi, T. Barklow, N. Barlow, B. M. Barnett, R. M. Barnett, A. Baroncelli, G. Barone, A. J. Barr, F. Barreiro, J. Barreiro Guimarães da Costa, P. Barrillon, R. Bartoldus, A. E. Barton, V. Bartsch, R. L. Bates, L. Batkova, J. R. Batley, A. Battaglia, M. Battistin, F. Bauer, H. S. Bawa, S. Beale, T. Beau, P. H. Beauchemin, R. Beccherle, P. Bechtle, H. P. Beck, A. K. Becker, S. Becker, M. Beckingham, K. H. Becks, A. J. Beddall, A. Beddall, S. Bedikian, V. A. Bednyakov, C. P. Bee, L. J. Beemster, M. Begel, S. Behar Harpaz, M. Beimforde, C. Belanger-Champagne, P. J. Bell, W. H. Bell, G. Bella, L. Bellagamba, F. Bellina, M. Bellomo, A. Belloni, O. Beloborodova, K. Belotskiy, O. Beltramello, O. Benary, D. Benchekroun, K. Bendtz, N. Benekos, Y. Benhammou, E. Benhar Noccioli, J. A. Benitez Garcia, D. P. Benjamin, M. Benoit, J. R. Bensinger, K. Benslama, S. Bentvelsen, D. Berge, E. Bergeaas Kuutmann, N. Berger, F. Berghaus, E. Berglund, J. Beringer, P. Bernat, R. Bernhard, C. Bernius, T. Berry, C. Bertella, A. Bertin, F. Bertolucci, M. I. Besana, G. J. Besjes, N. Besson, S. Bethke, W. Bhimji, R. M. Bianchi, M. Bianco, O. Biebel, S. P. Bieniek, K. Bierwagen, J. Biesiada, M. Biglietti, H. Bilokon, M. Bindi, S. Binet, A. Bingul, C. Bini, C. Biscarat, U. Bitenc, K. M. Black, R. E. Blair, J.-B. Blanchard, G. Blanchot, T. Blazek, C. Blocker, J. Blocki, A. Blondel, W. Blum, U. Blumenschein, G. J. Bobbink, V. B. Bobrovnikov, S. S. Bocchetta, A. Bocci, C. R. Boddy, M. Boehler, J. Boek, N. Boelaert, J. A. Bogaerts, A. Bogdanchikov, A. Bogouch, C. Bohm, J. Bohm, V. Boisvert, T. Bold, V. Boldea, N. M. Bolnet, M. Bomben, M. Bona, M. Boonekamp, C. N. Booth, S. Bordoni, C. Borer, A. Borisov, G. Borissov, I. Borjanovic, M. Borri, S. Borroni, V. Bortolotto, K. Bos, D. Boscherini, M. Bosman, H. Boterenbrood, J. Bouchami, J. Boudreau, E. V. Bouhova-Thacker, D. Boumediene, C. Bourdarios, N. Bousson, A. Boveia, J. Boyd, I. R. Boyko, I. Bozovic-Jelisavcic, J. Bracinik, P. Branchini, A. Brandt, G. Brandt, O. Brandt, U. Bratzler, B. Brau, J. E. Brau, H. M. Braun, S. F. Brazzale, B. Brelier, J. Bremer, K. Brendlinger, R. Brenner, S. Bressler, D. Britton, F. M. Brochu, I. Brock, R. Brock, F. Broggi, C. Bromberg, J. Bronner, G. Brooijmans, T. Brooks, W. K. Brooks, G. Brown, H. Brown, P. A. Bruckman de Renstrom, D. Bruncko, R. Bruneliere, S. Brunet, A. Bruni, G. Bruni, M. Bruschi, T. Buanes, Q. Buat, F. Bucci, J. Buchanan, P. Buchholz, R. M. Buckingham, A. G. Buckley, S. I. Buda, I. A. Budagov, B. Budick, V. Büscher, L. Bugge, O. Bulekov, A. C. Bundock, M. Bunse, T. Buran, H. Burckhart, S. Burdin, T. Burgess, S. Burke, E. Busato, P. Bussey, C. P. Buszello, B. Butler, J. M. Butler, C. M. Buttar, J. M. Butterworth, W. Buttinger, M. Byszewski, S. Cabrera Urbán, D. Caforio, O. Cakir, P. Calafiura, G. Calderini, P. Calfayan, R. Calkins, L. P. Caloba, R. Caloi, D. Calvet, S. Calvet, R. Camacho Toro, P. Camarri, D. Cameron, L. M. Caminada, S. Campana, M. Campanelli, V. Canale, F. Canelli, A. Canepa, J. Cantero, R. Cantrill, L. Capasso, M. D. M. Capeans Garrido, I. Caprini, M. Caprini, D. Capriotti, M. Capua, R. Caputo, R. Cardarelli, T. Carli, G. Carlino, L. Carminati, B. Caron, S. Caron, E. Carquin, G. D. Carrillo Montoya, A. A. Carter, J. R. Carter, J. Carvalho, D. Casadei, M. P. Casado, M. Cascella, C. Caso, A. M. Castaneda Hernandez, E. Castaneda-Miranda, V. Castillo Gimenez, N. F. Castro, G. Cataldi, P. Catastini, A. Catinaccio, J. R. Catmore, A. Cattai, G. Cattani, S. Caughron, V. Cavaliere, P. Cavalleri, D. Cavalli, M. Cavalli-Sforza, V. Cavasinni, F. Ceradini, A. S. Cerqueira, A. Cerri, L. Cerrito, F. Cerutti, S. A. Cetin, A. Chafaq, D. Chakraborty, I. Chalupkova, K. Chan, B. Chapleau, J. D. Chapman, J. W. Chapman, E. Chareyre, D. G. Charlton, V. Chavda, C. A. Chavez Barajas, S. Cheatham, S. Chekanov, S. V. Chekulaev, G. A. Chelkov, M. A. Chelstowska, C. Chen, H. Chen, S. Chen, X. Chen, Y. Chen, A. Cheplakov, R. Cherkaoui El Moursli, V. Chernyatin, E. Cheu, S. L. Cheung, L. Chevalier, G. Chiefari, L. Chikovani, J. T. Childers, A. Chilingarov, G. Chiodini, A. S. Chisholm, R. T. Chislett, A. Chitan, M. V. Chizhov, G. Choudalakis, S. Chouridou, I. A. Christidi, A. Christov, D. Chromek-Burckhart, M. L. Chu, J. Chudoba, G. Ciapetti, A. K. Ciftci, R. Ciftci, D. Cinca, V. Cindro, C. Ciocca, A. Ciocio, M. Cirilli, P. Cirkovic, M. Citterio, M. Ciubancan, A. Clark, P. J. Clark, R. N. Clarke, W. Cleland, J. C. Clemens, B. Clement, C. Clement, Y. Coadou, M. Cobal, A. Coccaro, J. Cochran, J. G. Cogan, J. Coggeshall, E. Cogneras, J. Colas, S. Cole, A. P. Colijn, N. J. Collins, C. Collins-Tooth, J. Collot, T. Colombo, G. Colon, P. Conde Muiño, E. Coniavitis, M. C. Conidi, S. M. Consonni, V. Consorti, S. Constantinescu, C. Conta, G. Conti, F. Conventi, M. Cooke, B. D. Cooper, A. M. Cooper-Sarkar, K. Copic, T. Cornelissen, M. Corradi, F. Corriveau, A. Cortes-Gonzalez, G. Cortiana, G. Costa, M. J. Costa, D. Costanzo, T. Costin, D. Côté, L. Courneyea, G. Cowan, C. Cowden, B. E. Cox, K. Cranmer, F. Crescioli, M. Cristinziani, G. Crosetti, S. Crépé-Renaudin, C.-M. Cuciuc, C. Cuenca Almenar, T. Cuhadar Donszelmann, M. Curatolo, C. J. Curtis, C. Cuthbert, P. Cwetanski, H. Czirr, P. Czodrowski, Z. Czyczula, S. D’Auria, M. D’Onofrio, A. D’Orazio, M. J. Da Cunha Sargedas De Sousa, C. Da Via, W. Dabrowski, A. Dafinca, T. Dai, C. Dallapiccola, M. Dam, M. Dameri, D. S. Damiani, H. O. Danielsson, V. Dao, G. Darbo, G. L. Darlea, J. A. Dassoulas, W. Davey, T. Davidek, N. Davidson, R. Davidson, E. Davies, M. Davies, O. Davignon, A. R. Davison, Y. Davygora, E. Dawe, I. Dawson, R. K. Daya-Ishmukhametova, K. De, R. de Asmundis, S. De Castro, S. De Cecco, J. de Graat, N. De Groot, P. de Jong, C. De La Taille, H. De la Torre, F. De Lorenzi, L. de Mora, L. De Nooij, D. De Pedis, A. De Salvo, U. De Sanctis, A. De Santo, J. B. De Vivie De Regie, G. De Zorzi, W. J. Dearnaley, R. Debbe, C. Debenedetti, B. Dechenaux, D. V. Dedovich, J. Degenhardt, C. Del Papa, J. Del Peso, T. Del Prete, T. Delemontex, M. Deliyergiyev, A. Dell’Acqua, L. Dell’Asta, M. Della Pietra, D. della Volpe, M. Delmastro, P. A. Delsart, C. Deluca, S. Demers, M. Demichev, B. Demirkoz, J. Deng, S. P. Denisov, D. Derendarz, J. E. Derkaoui, F. Derue, P. Dervan, K. Desch, E. Devetak, P. O. Deviveiros, A. Dewhurst, B. DeWilde, S. Dhaliwal, R. Dhullipudi, A. Di Ciaccio, L. Di Ciaccio, A. Di Girolamo, B. Di Girolamo, S. Di Luise, A. Di Mattia, B. Di Micco, R. Di Nardo, A. Di Simone, R. Di Sipio, M. A. Diaz, E. B. Diehl, J. Dietrich, T. A. Dietzsch, S. Diglio, K. Dindar Yagci, J. Dingfelder, F. Dinut, C. Dionisi, P. Dita, S. Dita, F. Dittus, F. Djama, T. Djobava, M. A. B. do Vale, A. Do Valle Wemans, T. K. O. Doan, M. Dobbs, R. Dobinson, D. Dobos, E. Dobson, J. Dodd, C. Doglioni, T. Doherty, Y. Doi, J. Dolejsi, I. Dolenc, Z. Dolezal, B. A. Dolgoshein, T. Dohmae, M. Donadelli, J. Donini, J. Dopke, A. Doria, A. Dos Anjos, A. Dotti, M. T. Dova, A. D. Doxiadis, A. T. Doyle, M. Dris, J. Dubbert, S. Dube, E. Duchovni, G. Duckeck, A. Dudarev, F. Dudziak, M. Dührssen, I. P. Duerdoth, L. Duflot, M-A. Dufour, L. Duguid, M. Dunford, H. Duran Yildiz, R. Duxfield, M. Dwuznik, F. Dydak, M. Düren, J. Ebke, S. Eckweiler, K. Edmonds, W. Edson, C. A. Edwards, N. C. Edwards, W. Ehrenfeld, T. Eifert, G. Eigen, K. Einsweiler, E. Eisenhandler, T. Ekelof, M. El Kacimi, M. Ellert, S. Elles, F. Ellinghaus, K. Ellis, N. Ellis, J. Elmsheuser, M. Elsing, D. Emeliyanov, R. Engelmann, A. Engl, B. Epp, J. Erdmann, A. Ereditato, D. Eriksson, J. Ernst, M. Ernst, J. Ernwein, D. Errede, S. Errede, E. Ertel, M. Escalier, H. Esch, C. Escobar, X. Espinal Curull, B. Esposito, F. Etienne, A. I. Etienvre, E. Etzion, D. Evangelakou, H. Evans, L. Fabbri, C. Fabre, R. M. Fakhrutdinov, S. Falciano, Y. Fang, M. Fanti, A. Farbin, A. Farilla, J. Farley, T. Farooque, S. Farrell, S. M. Farrington, P. Farthouat, P. Fassnacht, D. Fassouliotis, B. Fatholahzadeh, A. Favareto, L. Fayard, S. Fazio, R. Febbraro, P. Federic, O. L. Fedin, W. Fedorko, M. Fehling-Kaschek, L. Feligioni, D. Fellmann, C. Feng, E. J. Feng, A. B. Fenyuk, J. Ferencei, W. Fernando, S. Ferrag, J. Ferrando, V. Ferrara, A. Ferrari, P. Ferrari, R. Ferrari, D. E. Ferreira de Lima, A. Ferrer, D. Ferrere, C. Ferretti, A. Ferretto Parodi, M. Fiascaris, F. Fiedler, A. Filipčič, F. Filthaut, M. Fincke-Keeler, M. C. N. Fiolhais, L. Fiorini, A. Firan, G. Fischer, M. J. Fisher, M. Flechl, I. Fleck, J. Fleckner, P. Fleischmann, S. Fleischmann, T. Flick, A. Floderus, L. R. Flores Castillo, M. J. Flowerdew, T. Fonseca Martin, A. Formica, A. Forti, D. Fortin, D. Fournier, H. Fox, P. Francavilla, M. Franchini, S. Franchino, D. Francis, T. Frank, S. Franz, M. Fraternali, S. Fratina, S. T. French, C. Friedrich, F. Friedrich, R. Froeschl, D. Froidevaux, J. A. Frost, C. Fukunaga, E. Fullana Torregrosa, B. G. Fulsom, J. Fuster, C. Gabaldon, O. Gabizon, T. Gadfort, S. Gadomski, G. Gagliardi, P. Gagnon, C. Galea, E. J. Gallas, V. Gallo, B. J. Gallop, P. Gallus, K. K. Gan, Y. S. Gao, A. Gaponenko, F. Garberson, M. Garcia-Sciveres, C. García, J. E. García Navarro, R. W. Gardner, N. Garelli, H. Garitaonandia, V. Garonne, C. Gatti, G. Gaudio, B. Gaur, L. Gauthier, P. Gauzzi, I. L. Gavrilenko, C. Gay, G. Gaycken, E. N. Gazis, P. Ge, Z. Gecse, C. N. P. Gee, D. A. A. Geerts, Ch. Geich-Gimbel, K. Gellerstedt, C. Gemme, A. Gemmell, M. H. Genest, S. Gentile, M. George, S. George, P. Gerlach, A. Gershon, C. Geweniger, H. Ghazlane, N. Ghodbane, B. Giacobbe, S. Giagu, V. Giakoumopoulou, V. Giangiobbe, F. Gianotti, B. Gibbard, A. Gibson, S. M. Gibson, D. Gillberg, A. R. Gillman, D. M. Gingrich, J. Ginzburg, N. Giokaris, M. P. Giordani, R. Giordano, F. M. Giorgi, P. Giovannini, P. F. Giraud, D. Giugni, M. Giunta, P. Giusti, B. K. Gjelsten, L. K. Gladilin, C. Glasman, J. Glatzer, A. Glazov, K. W. Glitza, G. L. Glonti, J. R. Goddard, J. Godfrey, J. Godlewski, M. Goebel, T. Göpfert, C. Goeringer, C. Gössling, S. Goldfarb, T. Golling, A. Gomes, L. S. Gomez Fajardo, R. Gonçalo, J. Goncalves Pinto Firmino Da Costa, L. Gonella, S. Gonzalez, S. González de la Hoz, G. Gonzalez Parra, M. L. Gonzalez Silva, S. Gonzalez-Sevilla, J. J. Goodson, L. Goossens, P. A. Gorbounov, H. A. Gordon, I. Gorelov, G. Gorfine, B. Gorini, E. Gorini, A. Gorišek, E. Gornicki, B. Gosdzik, A. T. Goshaw, M. Gosselink, M. I. Gostkin, I. Gough Eschrich, M. Gouighri, D. Goujdami, M. P. Goulette, A. G. Goussiou, C. Goy, S. Gozpinar, I. Grabowska-Bold, P. Grafström, K-J. Grahn, F. Grancagnolo, S. Grancagnolo, V. Grassi, V. Gratchev, N. Grau, H. M. Gray, J. A. Gray, E. Graziani, O. G. Grebenyuk, T. Greenshaw, Z. D. Greenwood, K. Gregersen, I. M. Gregor, P. Grenier, J. Griffiths, N. Grigalashvili, A. A. Grillo, S. Grinstein, Y. V. Grishkevich, J.-F. Grivaz, E. Gross, J. Grosse-Knetter, J. Groth-Jensen, K. Grybel, D. Guest, C. Guicheney, S. Guindon, U. Gul, H. Guler, J. Gunther, B. Guo, J. Guo, P. Gutierrez, N. Guttman, O. Gutzwiller, C. Guyot, C. Gwenlan, C. B. Gwilliam, A. Haas, S. Haas, C. Haber, H. K. Hadavand, D. R. Hadley, P. Haefner, F. Hahn, S. Haider, Z. Hajduk, H. Hakobyan, D. Hall, J. Haller, K. Hamacher, P. Hamal, M. Hamer, A. Hamilton, S. Hamilton, L. Han, K. Hanagaki, K. Hanawa, M. Hance, C. Handel, P. Hanke, J. R. Hansen, J. B. Hansen, J. D. Hansen, P. H. Hansen, P. Hansson, K. Hara, G. A. Hare, T. Harenberg, S. Harkusha, D. Harper, R. D. Harrington, O. M. Harris, J. Hartert, F. Hartjes, T. Haruyama, A. Harvey, S. Hasegawa, Y. Hasegawa, S. Hassani, S. Haug, M. Hauschild, R. Hauser, M. Havranek, C. M. Hawkes, R. J. Hawkings, A. D. Hawkins, D. Hawkins, T. Hayakawa, T. Hayashi, D. Hayden, C. P. Hays, H. S. Hayward, S. J. Haywood, M. He, S. J. Head, V. Hedberg, L. Heelan, S. Heim, B. Heinemann, S. Heisterkamp, L. Helary, C. Heller, M. Heller, S. Hellman, D. Hellmich, C. Helsens, R. C. W. Henderson, M. Henke, A. Henrichs, A. M. Henriques Correia, S. Henrot-Versille, C. Hensel, T. Henß, C. M. Hernandez, Y. Hernández Jiménez, R. Herrberg, G. Herten, R. Hertenberger, L. Hervas, G. G. Hesketh, N. P. Hessey, E. Higón-Rodriguez, J. C. Hill, K. H. Hiller, S. Hillert, S. J. Hillier, I. Hinchliffe, E. Hines, M. Hirose, F. Hirsch, D. Hirschbuehl, J. Hobbs, N. Hod, M. C. Hodgkinson, P. Hodgson, A. Hoecker, M. R. Hoeferkamp, J. Hoffman, D. Hoffmann, M. Hohlfeld, M. Holder, S. O. Holmgren, T. Holy, J. L. Holzbauer, T. M. Hong, L. Hooft van Huysduynen, C. Horn, S. Horner, J-Y. Hostachy, S. Hou, A. Hoummada, J. Howard, J. Howarth, I. Hristova, J. Hrivnac, T. Hryn’ova, P. J. Hsu, S.-C. Hsu, Z. Hubacek, F. Hubaut, F. Huegging, A. Huettmann, T. B. Huffman, E. W. Hughes, G. Hughes, M. Huhtinen, M. Hurwitz, U. Husemann, N. Huseynov, J. Huston, J. Huth, G. Iacobucci, G. Iakovidis, M. Ibbotson, I. Ibragimov, L. Iconomidou-Fayard, J. Idarraga, P. Iengo, O. Igonkina, Y. Ikegami, M. Ikeno, D. Iliadis, N. Ilic, T. Ince, J. Inigo-Golfin, P. Ioannou, M. Iodice, K. Iordanidou, V. Ippolito, A. Irles Quiles, C. Isaksson, M. Ishino, M. Ishitsuka, R. Ishmukhametov, C. Issever, S. Istin, A. V. Ivashin, W. Iwanski, H. Iwasaki, J. M. Izen, V. Izzo, B. Jackson, J. N. Jackson, P. Jackson, M. R. Jaekel, V. Jain, K. Jakobs, S. Jakobsen, T. Jakoubek, J. Jakubek, D. K. Jana, E. Jansen, H. Jansen, A. Jantsch, M. Janus, G. Jarlskog, L. Jeanty, I. Jen-La Plante, D. Jennens, P. Jenni, A. E. Loevschall-Jensen, P. Jež, S. Jézéquel, M. K. Jha, H. Ji, W. Ji, J. Jia, Y. Jiang, M. Jimenez Belenguer, S. Jin, O. Jinnouchi, M. D. Joergensen, D. Joffe, M. Johansen, K. E. Johansson, P. Johansson, S. Johnert, K. A. Johns, K. Jon-And, G. Jones, R. W. L. Jones, T. J. Jones, C. Joram, P. M. Jorge, K. D. Joshi, J. Jovicevic, T. Jovin, X. Ju, C. A. Jung, R. M. Jungst, V. Juranek, P. Jussel, A. Juste Rozas, S. Kabana, M. Kaci, A. Kaczmarska, P. Kadlecik, M. Kado, H. Kagan, M. Kagan, E. Kajomovitz, S. Kalinin, L. V. Kalinovskaya, S. Kama, N. Kanaya, M. Kaneda, S. Kaneti, T. Kanno, V. A. Kantserov, J. Kanzaki, B. Kaplan, A. Kapliy, J. Kaplon, D. Kar, M. Karagounis, K. Karakostas, M. Karnevskiy, V. Kartvelishvili, A. N. Karyukhin, L. Kashif, G. Kasieczka, R. D. Kass, A. Kastanas, M. Kataoka, Y. Kataoka, E. Katsoufis, J. Katzy, V. Kaushik, K. Kawagoe, T. Kawamoto, G. Kawamura, M. S. Kayl, S. Kazama, V. A. Kazanin, M. Y. Kazarinov, R. Keeler, R. Kehoe, M. Keil, G. D. Kekelidze, J. S. Keller, M. Kenyon, O. Kepka, N. Kerschen, B. P. Kerševan, S. Kersten, K. Kessoku, J. Keung, F. Khalil-zada, H. Khandanyan, A. Khanov, D. Kharchenko, A. Khodinov, A. Khomich, T. J. Khoo, G. Khoriauli, A. Khoroshilov, V. Khovanskiy, E. Khramov, J. Khubua, H. Kim, S. H. Kim, N. Kimura, O. Kind, B. T. King, M. King, R. S. B. King, J. Kirk, A. E. Kiryunin, T. Kishimoto, D. Kisielewska, T. Kitamura, T. Kittelmann, E. Kladiva, M. Klein, U. Klein, K. Kleinknecht, M. Klemetti, A. Klier, P. Klimek, A. Klimentov, R. Klingenberg, J. A. Klinger, E. B. Klinkby, T. Klioutchnikova, P. F. Klok, S. Klous, E.-E. Kluge, T. Kluge, P. Kluit, S. Kluth, N. S. Knecht, E. Kneringer, E. B. F. G. Knoops, A. Knue, B. R. Ko, T. Kobayashi, M. Kobel, M. Kocian, P. Kodys, K. Köneke, A. C. König, S. Koenig, L. Köpke, F. Koetsveld, P. Koevesarki, T. Koffas, E. Koffeman, L. A. Kogan, S. Kohlmann, F. Kohn, Z. Kohout, T. Kohriki, T. Koi, G. M. Kolachev, H. Kolanoski, V. Kolesnikov, I. Koletsou, J. Koll, M. Kollefrath, A. A. Komar, Y. Komori, T. Kondo, T. Kono, A. I. Kononov, R. Konoplich, N. Konstantinidis, S. Koperny, K. Korcyl, K. Kordas, A. Korn, A. Korol, I. Korolkov, E. V. Korolkova, V. A. Korotkov, O. Kortner, S. Kortner, V. V. Kostyukhin, S. Kotov, V. M. Kotov, A. Kotwal, C. Kourkoumelis, V. Kouskoura, A. Koutsman, R. Kowalewski, T. Z. Kowalski, W. Kozanecki, A. S. Kozhin, V. Kral, V. A. Kramarenko, G. Kramberger, M. W. Krasny, A. Krasznahorkay, J. K. Kraus, S. Kreiss, F. Krejci, J. Kretzschmar, N. Krieger, P. Krieger, K. Kroeninger, H. Kroha, J. Kroll, J. Kroseberg, J. Krstic, U. Kruchonak, H. Krüger, T. Kruker, N. Krumnack, Z. V. Krumshteyn, T. Kubota, S. Kuday, S. Kuehn, A. Kugel, T. Kuhl, D. Kuhn, V. Kukhtin, Y. Kulchitsky, S. Kuleshov, C. Kummer, M. Kuna, J. Kunkle, A. Kupco, H. Kurashige, M. Kurata, Y. A. Kurochkin, V. Kus, E. S. Kuwertz, M. Kuze, J. Kvita, R. Kwee, A. La Rosa, L. La Rotonda, L. Labarga, J. Labbe, S. Lablak, C. Lacasta, F. Lacava, H. Lacker, D. Lacour, V. R. Lacuesta, E. Ladygin, R. Lafaye, B. Laforge, T. Lagouri, S. Lai, E. Laisne, M. Lamanna, L. Lambourne, C. L. Lampen, W. Lampl, E. Lancon, U. Landgraf, M. P. J. Landon, J. L. Lane, V. S. Lang, C. Lange, A. J. Lankford, F. Lanni, K. Lantzsch, S. Laplace, C. Lapoire, J. F. Laporte, T. Lari, A. Larner, M. Lassnig, P. Laurelli, V. Lavorini, W. Lavrijsen, P. Laycock, O. Le Dortz, E. Le Guirriec, C. Le Maner, E. Le Menedeu, T. LeCompte, F. Ledroit-Guillon, H. Lee, J. S. H. Lee, S. C. Lee, L. Lee, M. Lefebvre, M. Legendre, F. Legger, C. Leggett, M. Lehmacher, G. Lehmann Miotto, X. Lei, M. A. L. Leite, R. Leitner, D. Lellouch, B. Lemmer, V. Lendermann, K. J. C. Leney, T. Lenz, G. Lenzen, B. Lenzi, K. Leonhardt, S. Leontsinis, F. Lepold, C. Leroy, J-R. Lessard, C. G. Lester, C. M. Lester, J. Levêque, D. Levin, L. J. Levinson, A. Lewis, G. H. Lewis, A. M. Leyko, M. Leyton, B. Li, H. Li, S. Li, X. Li, Z. Liang, H. Liao, B. Liberti, P. Lichard, M. Lichtnecker, K. Lie, W. Liebig, C. Limbach, A. Limosani, M. Limper, S. C. Lin, F. Linde, J. T. Linnemann, E. Lipeles, A. Lipniacka, T. M. Liss, D. Lissauer, A. Lister, A. M. Litke, C. Liu, D. Liu, H. Liu, J. B. Liu, L. Liu, M. Liu, Y. Liu, M. Livan, S. S. A. Livermore, A. Lleres, J. Llorente Merino, S. L. Lloyd, E. Lobodzinska, P. Loch, W. S. Lockman, T. Loddenkoetter, F. K. Loebinger, A. Loginov, C. W. Loh, T. Lohse, K. Lohwasser, M. Lokajicek, V. P. Lombardo, R. E. Long, L. Lopes, D. Lopez Mateos, J. Lorenz, N. Lorenzo Martinez, M. Losada, P. Loscutoff, F. Lo Sterzo, M. J. Losty, X. Lou, A. Lounis, K. F. Loureiro, J. Love, P. A. Love, A. J. Lowe, F. Lu, H. J. Lubatti, C. Luci, A. Lucotte, A. Ludwig, D. Ludwig, I. Ludwig, J. Ludwig, F. Luehring, G. Luijckx, W. Lukas, D. Lumb, L. Luminari, E. Lund, B. Lund-Jensen, B. Lundberg, J. Lundberg, O. Lundberg, J. Lundquist, M. Lungwitz, D. Lynn, E. Lytken, H. Ma, L. L. Ma, G. Maccarrone, A. Macchiolo, B. Maček, J. Machado Miguens, R. Mackeprang, R. J. Madaras, H. J. Maddocks, W. F. Mader, R. Maenner, T. Maeno, P. Mättig, S. Mättig, L. Magnoni, E. Magradze, K. Mahboubi, S. Mahmoud, G. Mahout, C. Maiani, C. Maidantchik, A. Maio, S. Majewski, Y. Makida, N. Makovec, P. Mal, B. Malaescu, Pa. Malecki, P. Malecki, V. P. Maleev, F. Malek, U. Mallik, D. Malon, C. Malone, S. Maltezos, V. Malyshev, S. Malyukov, R. Mameghani, J. Mamuzic, A. Manabe, L. Mandelli, I. Mandić, R. Mandrysch, J. Maneira, P. S. Mangeard, L. Manhaes de Andrade Filho, J. A. Manjarres Ramos, A. Mann, P. M. Manning, A. Manousakis-Katsikakis, B. Mansoulie, A. Mapelli, L. Mapelli, L. March, J. F. Marchand, F. Marchese, G. Marchiori, M. Marcisovsky, C. P. Marino, F. Marroquim, Z. Marshall, F. K. Martens, L. F. Marti, S. Marti-Garcia, B. Martin, B. Martin, J. P. Martin, T. A. Martin, V. J. Martin, B. Martin dit Latour, S. Martin-Haugh, M. Martinez, V. Martinez Outschoorn, A. C. Martyniuk, M. Marx, F. Marzano, A. Marzin, L. Masetti, T. Mashimo, R. Mashinistov, J. Masik, A. L. Maslennikov, I. Massa, G. Massaro, N. Massol, P. Mastrandrea, A. Mastroberardino, T. Masubuchi, P. Matricon, H. Matsunaga, T. Matsushita, C. Mattravers, J. Maurer, S. J. Maxfield, A. Mayne, R. Mazini, M. Mazur, L. Mazzaferro, M. Mazzanti, S. P. Mc Kee, A. McCarn, R. L. McCarthy, T. G. McCarthy, N. A. McCubbin, K. W. McFarlane, J. A. Mcfayden, G. Mchedlidze, T. Mclaughlan, S. J. McMahon, R. A. McPherson, A. Meade, J. Mechnich, M. Mechtel, M. Medinnis, R. Meera-Lebbai, T. Meguro, R. Mehdiyev, S. Mehlhase, A. Mehta, K. Meier, B. Meirose, C. Melachrinos, B. R. Mellado Garcia, F. Meloni, L. Mendoza Navas, Z. Meng, A. Mengarelli, S. Menke, E. Meoni, K. M. Mercurio, P. Mermod, L. Merola, C. Meroni, F. S. Merritt, H. Merritt, A. Messina, J. Metcalfe, A. S. Mete, C. Meyer, C. Meyer, J-P. Meyer, J. Meyer, J. Meyer, T. C. Meyer, J. Miao, S. Michal, L. Micu, R. P. Middleton, S. Migas, L. Mijović, G. Mikenberg, M. Mikestikova, M. Mikuž, D. W. Miller, R. J. Miller, W. J. Mills, C. Mills, A. Milov, D. A. Milstead, D. Milstein, A. A. Minaenko, M. Miñano Moya, I. A. Minashvili, A. I. Mincer, B. Mindur, M. Mineev, Y. Ming, L. M. Mir, G. Mirabelli, J. Mitrevski, V. A. Mitsou, S. Mitsui, P. S. Miyagawa, J. U. Mjörnmark, T. Moa, V. Moeller, K. Mönig, N. Möser, S. Mohapatra, W. Mohr, R. Moles-Valls, J. Monk, E. Monnier, J. Montejo Berlingen, F. Monticelli, S. Monzani, R. W. Moore, G. F. Moorhead, C. Mora Herrera, A. Moraes, N. Morange, J. Morel, G. Morello, D. Moreno, M. Moreno Llácer, P. Morettini, M. Morgenstern, M. Morii, A. K. Morley, G. Mornacchi, J. D. Morris, L. Morvaj, H. G. Moser, M. Mosidze, J. Moss, R. Mount, E. Mountricha, S. V. Mouraviev, E. J. W. Moyse, F. Mueller, J. Mueller, K. Mueller, T. A. Müller, T. Mueller, D. Muenstermann, Y. Munwes, W. J. Murray, I. Mussche, E. Musto, A. G. Myagkov, M. Myska, J. Nadal, K. Nagai, R. Nagai, K. Nagano, A. Nagarkar, Y. Nagasaka, M. Nagel, A. M. Nairz, Y. Nakahama, K. Nakamura, T. Nakamura, I. Nakano, G. Nanava, A. Napier, R. Narayan, M. Nash, T. Nattermann, T. Naumann, G. Navarro, H. A. Neal, P. Yu. Nechaeva, T. J. Neep, A. Negri, G. Negri, M. Negrini, S. Nektarijevic, A. Nelson, T. K. Nelson, S. Nemecek, P. Nemethy, A. A. Nepomuceno, M. Nessi, M. S. Neubauer, M. Neumann, A. Neusiedl, R. M. Neves, P. Nevski, P. R. Newman, V. Nguyen Thi Hong, R. B. Nickerson, R. Nicolaidou, B. Nicquevert, F. Niedercorn, J. Nielsen, N. Nikiforou, A. Nikiforov, V. Nikolaenko, I. Nikolic-Audit, K. Nikolics, K. Nikolopoulos, H. Nilsen, P. Nilsson, Y. Ninomiya, A. Nisati, R. Nisius, T. Nobe, L. Nodulman, M. Nomachi, I. Nomidis, S. Norberg, M. Nordberg, P. R. Norton, J. Novakova, M. Nozaki, L. Nozka, I. M. Nugent, A.-E. Nuncio-Quiroz, G. Nunes Hanninger, T. Nunnemann, E. Nurse, B. J. O’Brien, S. W. O’Neale, D. C. O’Neil, V. O’Shea, L. B. Oakes, F. G. Oakham, H. Oberlack, J. Ocariz, A. Ochi, S. Oda, S. Odaka, J. Odier, H. Ogren, A. Oh, S. H. Oh, C. C. Ohm, T. Ohshima, H. Okawa, Y. Okumura, T. Okuyama, A. Olariu, A. G. Olchevski, S. A. Olivares Pino, M. Oliveira, D. Oliveira Damazio, E. Oliver Garcia, D. Olivito, A. Olszewski, J. Olszowska, A. Onofre, P. U. E. Onyisi, C. J. Oram, M. J. Oreglia, Y. Oren, D. Orestano, N. Orlando, I. Orlov, C. Oropeza Barrera, R. S. Orr, B. Osculati, R. Ospanov, C. Osuna, G. Otero y Garzon, J. P. Ottersbach, M. Ouchrif, E. A. Ouellette, F. Ould-Saada, A. Ouraou, Q. Ouyang, A. Ovcharova, M. Owen, S. Owen, V. E. Ozcan, N. Ozturk, A. Pacheco Pages, C. Padilla Aranda, S. Pagan Griso, E. Paganis, C. Pahl, F. Paige, P. Pais, K. Pajchel, G. Palacino, C. P. Paleari, S. Palestini, D. Pallin, A. Palma, J. D. Palmer, Y. B. Pan, E. Panagiotopoulou, P. Pani, N. Panikashvili, S. Panitkin, D. Pantea, A. Papadelis, Th. D. Papadopoulou, A. Paramonov, D. Paredes Hernandez, W. Park, M. A. Parker, F. Parodi, J. A. Parsons, U. Parzefall, S. Pashapour, E. Pasqualucci, S. Passaggio, A. Passeri, F. Pastore, Fr. Pastore, G. Pásztor, S. Pataraia, N. Patel, J. R. Pater, S. Patricelli, T. Pauly, M. Pecsy, S. Pedraza Lopez, M. I. Pedraza Morales, S. V. Peleganchuk, D. Pelikan, H. Peng, B. Penning, A. Penson, J. Penwell, M. Perantoni, K. Perez, T. Perez Cavalcanti, E. Perez Codina, M. T. Pérez García-Estañ, V. Perez Reale, L. Perini, H. Pernegger, R. Perrino, P. Perrodo, V. D. Peshekhonov, K. Peters, B. A. Petersen, J. Petersen, T. C. Petersen, E. Petit, A. Petridis, C. Petridou, E. Petrolo, F. Petrucci, D. Petschull, M. Petteni, R. Pezoa, A. Phan, P. W. Phillips, G. Piacquadio, A. Picazio, E. Piccaro, M. Piccinini, S. M. Piec, R. Piegaia, D. T. Pignotti, J. E. Pilcher, A. D. Pilkington, J. Pina, M. Pinamonti, A. Pinder, J. L. Pinfold, B. Pinto, C. Pizio, M. Plamondon, M.-A. Pleier, E. Plotnikova, A. Poblaguev, S. Poddar, F. Podlyski, L. Poggioli, M. Pohl, G. Polesello, A. Policicchio, A. Polini, J. Poll, V. Polychronakos, D. Pomeroy, K. Pommès, L. Pontecorvo, B. G. Pope, G. A. Popeneciu, D. S. Popovic, A. Poppleton, X. Portell Bueso, G. E. Pospelov, S. Pospisil, I. N. Potrap, C. J. Potter, C. T. Potter, G. Poulard, J. Poveda, V. Pozdnyakov, R. Prabhu, P. Pralavorio, A. Pranko, S. Prasad, R. Pravahan, S. Prell, K. Pretzl, D. Price, J. Price, L. E. Price, D. Prieur, M. Primavera, K. Prokofiev, F. Prokoshin, S. Protopopescu, J. Proudfoot, X. Prudent, M. Przybycien, H. Przysiezniak, S. Psoroulas, E. Ptacek, E. Pueschel, J. Purdham, M. Purohit, P. Puzo, Y. Pylypchenko, J. Qian, A. Quadt, D. R. Quarrie, W. B. Quayle, F. Quinonez, M. Raas, V. Radescu, P. Radloff, T. Rador, F. Ragusa, G. Rahal, A. M. Rahimi, D. Rahm, S. Rajagopalan, M. Rammensee, M. Rammes, A. S. Randle-Conde, K. Randrianarivony, F. Rauscher, T. C. Rave, M. Raymond, A. L. Read, D. M. Rebuzzi, A. Redelbach, G. Redlinger, R. Reece, K. Reeves, E. Reinherz-Aronis, A. Reinsch, H. Reisin, I. Reisinger, C. Rembser, Z. L. Ren, A. Renaud, M. Rescigno, S. Resconi, B. Resende, P. Reznicek, R. Rezvani, R. Richter, E. Richter-Was, M. Ridel, M. Rijpstra, M. Rijssenbeek, A. Rimoldi, L. Rinaldi, R. R. Rios, I. Riu, G. Rivoltella, F. Rizatdinova, E. Rizvi, S. H. Robertson, A. Robichaud-Veronneau, D. Robinson, J. E. M. Robinson, A. Robson, J. G. Rocha de Lima, C. Roda, D. Roda Dos Santos, A. Roe, S. Roe, O. Røhne, S. Rolli, A. Romaniouk, M. Romano, G. Romeo, E. Romero Adam, L. Roos, E. Ros, S. Rosati, K. Rosbach, A. Rose, M. Rose, G. A. Rosenbaum, E. I. Rosenberg, P. L. Rosendahl, O. Rosenthal, L. Rosselet, V. Rossetti, E. Rossi, L. P. Rossi, M. Rotaru, I. Roth, J. Rothberg, D. Rousseau, C. R. Royon, A. Rozanov, Y. Rozen, X. Ruan, F. Rubbo, I. Rubinskiy, N. Ruckstuhl, V. I. Rud, C. Rudolph, G. Rudolph, F. Rühr, A. Ruiz-Martinez, L. Rumyantsev, Z. Rurikova, N. A. Rusakovich, J. P. Rutherfoord, C. Ruwiedel, P. Ruzicka, Y. F. Ryabov, M. Rybar, G. Rybkin, N. C. Ryder, A. F. Saavedra, S. Sacerdoti, I. Sadeh, H. F-W. Sadrozinski, R. Sadykov, F. Safai Tehrani, H. Sakamoto, G. Salamanna, A. Salamon, M. Saleem, D. Salek, D. Salihagic, A. Salnikov, J. Salt, B. M. Salvachua Ferrando, D. Salvatore, F. Salvatore, A. Salvucci, A. Salzburger, D. Sampsonidis, B. H. Samset, A. Sanchez, V. Sanchez Martinez, H. Sandaker, H. G. Sander, M. P. Sanders, M. Sandhoff, T. Sandoval, C. Sandoval, R. Sandstroem, D. P. C. Sankey, A. Sansoni, C. Santamarina Rios, C. Santoni, R. Santonico, H. Santos, J. G. Saraiva, T. Sarangi, E. Sarkisyan-Grinbaum, F. Sarri, G. Sartisohn, O. Sasaki, Y. Sasaki, N. Sasao, I. Satsounkevitch, G. Sauvage, E. Sauvan, J. B. Sauvan, P. Savard, V. Savinov, D. O. Savu, L. Sawyer, D. H. Saxon, J. Saxon, C. Sbarra, A. Sbrizzi, D. A. Scannicchio, M. Scarcella, J. Schaarschmidt, P. Schacht, D. Schaefer, U. Schäfer, S. Schaepe, S. Schaetzel, A. C. Schaffer, D. Schaile, R. D. Schamberger, A. G. Schamov, V. Scharf, V. A. Schegelsky, D. Scheirich, M. Schernau, M. I. Scherzer, C. Schiavi, J. Schieck, M. Schioppa, S. Schlenker, E. Schmidt, K. Schmieden, C. Schmitt, S. Schmitt, M. Schmitz, B. Schneider, U. Schnoor, A. Schoening, A. L. S. Schorlemmer, M. Schott, D. Schouten, J. Schovancova, M. Schram, C. Schroeder, N. Schroer, M. J. Schultens, J. Schultes, H.-C. Schultz-Coulon, H. Schulz, M. Schumacher, B. A. Schumm, Ph. Schune, C. Schwanenberger, A. Schwartzman, Ph. Schwemling, R. Schwienhorst, R. Schwierz, J. Schwindling, T. Schwindt, M. Schwoerer, G. Sciolla, W. G. Scott, J. Searcy, G. Sedov, E. Sedykh, S. C. Seidel, A. Seiden, F. Seifert, J. M. Seixas, G. Sekhniaidze, S. J. Sekula, K. E. Selbach, D. M. Seliverstov, B. Sellden, G. Sellers, M. Seman, N. Semprini-Cesari, C. Serfon, L. Serin, L. Serkin, R. Seuster, H. Severini, A. Sfyrla, E. Shabalina, M. Shamim, L. Y. Shan, J. T. Shank, Q. T. Shao, M. Shapiro, P. B. Shatalov, K. Shaw, D. Sherman, P. Sherwood, A. Shibata, S. Shimizu, M. Shimojima, T. Shin, M. Shiyakova, A. Shmeleva, M. J. Shochet, D. Short, S. Shrestha, E. Shulga, M. A. Shupe, P. Sicho, A. Sidoti, F. Siegert, Dj. Sijacki, O. Silbert, J. Silva, Y. Silver, D. Silverstein, S. B. Silverstein, V. Simak, O. Simard, Lj. Simic, S. Simion, E. Simioni, B. Simmons, R. Simoniello, M. Simonyan, P. Sinervo, N. B. Sinev, V. Sipica, G. Siragusa, A. Sircar, A. N. Sisakyan, S. Yu. Sivoklokov, J. Sjölin, T. B. Sjursen, L. A. Skinnari, H. P. Skottowe, K. Skovpen, P. Skubic, M. Slater, T. Slavicek, K. Sliwa, V. Smakhtin, B. H. Smart, S. Yu. Smirnov, Y. Smirnov, L. N. Smirnova, O. Smirnova, B. C. Smith, D. Smith, K. M. Smith, M. Smizanska, K. Smolek, A. A. Snesarev, S. W. Snow, J. Snow, S. Snyder, R. Sobie, J. Sodomka, A. Soffer, C. A. Solans, M. Solar, J. Solc, E. Yu. Soldatov, U. Soldevila, E. Solfaroli Camillocci, A. A. Solodkov, O. V. Solovyanov, V. Solovyev, N. Soni, V. Sopko, B. Sopko, M. Sosebee, R. Soualah, A. Soukharev, S. Spagnolo, F. Spanò, R. Spighi, G. Spigo, R. Spiwoks, M. Spousta, T. Spreitzer, B. Spurlock, R. D. St. Denis, J. Stahlman, R. Stamen, E. Stanecka, R. W. Stanek, C. Stanescu, M. Stanescu-Bellu, S. Stapnes, E. A. Starchenko, J. Stark, P. Staroba, P. Starovoitov, R. Staszewski, A. Staude, P. Stavina, G. Steele, P. Steinbach, P. Steinberg, I. Stekl, B. Stelzer, H. J. Stelzer, O. Stelzer-Chilton, H. Stenzel, S. Stern, G. A. Stewart, J. A. Stillings, M. C. Stockton, K. Stoerig, G. Stoicea, S. Stonjek, P. Strachota, A. R. Stradling, A. Straessner, J. Strandberg, S. Strandberg, A. Strandlie, M. Strang, E. Strauss, M. Strauss, P. Strizenec, R. Ströhmer, D. M. Strom, J. A. Strong, R. Stroynowski, J. Strube, B. Stugu, I. Stumer, J. Stupak, P. Sturm, N. A. Styles, D. A. Soh, D. Su, HS. Subramania, A. Succurro, Y. Sugaya, C. Suhr, M. Suk, V. V. Sulin, S. Sultansoy, T. Sumida, X. Sun, J. E. Sundermann, K. Suruliz, G. Susinno, M. R. Sutton, Y. Suzuki, Y. Suzuki, M. Svatos, S. Swedish, I. Sykora, T. Sykora, J. Sánchez, D. Ta, K. Tackmann, A. Taffard, R. Tafirout, N. Taiblum, Y. Takahashi, H. Takai, R. Takashima, H. Takeda, T. Takeshita, Y. Takubo, M. Talby, A. Talyshev, M. C. Tamsett, J. Tanaka, R. Tanaka, S. Tanaka, S. Tanaka, A. J. Tanasijczuk, K. Tani, N. Tannoury, S. Tapprogge, D. Tardif, S. Tarem, F. Tarrade, G. F. Tartarelli, P. Tas, M. Tasevsky, E. Tassi, M. Tatarkhanov, Y. Tayalati, C. Taylor, F. E. Taylor, G. N. Taylor, W. Taylor, M. Teinturier, M. Teixeira Dias Castanheira, P. Teixeira-Dias, K. K. Temming, H. Ten Kate, P. K. Teng, S. Terada, K. Terashi, J. Terron, M. Testa, R. J. Teuscher, J. Therhaag, T. Theveneaux-Pelzer, S. Thoma, J. P. Thomas, E. N. Thompson, P. D. Thompson, P. D. Thompson, A. S. Thompson, L. A. Thomsen, E. Thomson, M. Thomson, W. M. Thong, R. P. Thun, F. Tian, M. J. Tibbetts, T. Tic, V. O. Tikhomirov, Y. A. Tikhonov, S. Timoshenko, P. Tipton, S. Tisserant, T. Todorov, S. Todorova-Nova, B. Toggerson, J. Tojo, S. Tokár, K. Tokushuku, K. Tollefson, M. Tomoto, L. Tompkins, K. Toms, A. Tonoyan, C. Topfel, N. D. Topilin, I. Torchiani, E. Torrence, H. Torres, E. Torró Pastor, J. Toth, F. Touchard, D. R. Tovey, T. Trefzger, L. Tremblet, A. Tricoli, I. M. Trigger, S. Trincaz-Duvoid, M. F. Tripiana, N. Triplett, W. Trischuk, B. Trocmé, C. Troncon, M. Trottier-McDonald, M. Trzebinski, A. Trzupek, C. Tsarouchas, J. C-L. Tseng, M. Tsiakiris, P. V. Tsiareshka, D. Tsionou, G. Tsipolitis, S. Tsiskaridze, V. Tsiskaridze, E. G. Tskhadadze, I. I. Tsukerman, V. Tsulaia, J.-W. Tsung, S. Tsuno, D. Tsybychev, A. Tua, A. Tudorache, V. Tudorache, J. M. Tuggle, M. Turala, D. Turecek, I. Turk Cakir, E. Turlay, R. Turra, P. M. Tuts, A. Tykhonov, M. Tylmad, M. Tyndel, G. Tzanakos, K. Uchida, I. Ueda, R. Ueno, M. Ugland, M. Uhlenbrock, M. Uhrmacher, F. Ukegawa, G. Unal, A. Undrus, G. Unel, Y. Unno, D. Urbaniec, G. Usai, M. Uslenghi, L. Vacavant, V. Vacek, B. Vachon, S. Vahsen, J. Valenta, S. Valentinetti, A. Valero, S. Valkar, E. Valladolid Gallego, S. Vallecorsa, J. A. Valls Ferrer, P. C. Van Der Deijl, R. van der Geer, H. van der Graaf, R. Van Der Leeuw, E. van der Poel, D. van der Ster, N. van Eldik, P. van Gemmeren, I. van Vulpen, M. Vanadia, W. Vandelli, A. Vaniachine, P. Vankov, F. Vannucci, R. Vari, T. Varol, D. Varouchas, A. Vartapetian, K. E. Varvell, V. I. Vassilakopoulos, F. Vazeille, T. Vazquez Schroeder, G. Vegni, J. J. Veillet, F. Veloso, R. Veness, S. Veneziano, A. Ventura, D. Ventura, M. Venturi, N. Venturi, V. Vercesi, M. Verducci, W. Verkerke, J. C. Vermeulen, A. Vest, M. C. Vetterli, I. Vichou, T. Vickey, O. E. Vickey Boeriu, G. H. A. Viehhauser, S. Viel, M. Villa, M. Villaplana Perez, E. Vilucchi, M. G. Vincter, E. Vinek, V. B. Vinogradov, M. Virchaux, J. Virzi, O. Vitells, M. Viti, I. Vivarelli, F. Vives Vaque, S. Vlachos, D. Vladoiu, M. Vlasak, A. Vogel, P. Vokac, G. Volpi, M. Volpi, G. Volpini, H. von der Schmitt, H. von Radziewski, E. von Toerne, V. Vorobel, V. Vorwerk, M. Vos, R. Voss, T. T. Voss, J. H. Vossebeld, N. Vranjes, M. Vranjes Milosavljevic, V. Vrba, M. Vreeswijk, T. Vu Anh, R. Vuillermet, I. Vukotic, W. Wagner, P. Wagner, H. Wahlen, S. Wahrmund, J. Wakabayashi, S. Walch, J. Walder, R. Walker, W. Walkowiak, R. Wall, P. Waller, B. Walsh, C. Wang, H. Wang, H. Wang, J. Wang, J. Wang, R. Wang, S. M. Wang, T. Wang, A. Warburton, C. P. Ward, M. Warsinsky, A. Washbrook, C. Wasicki, I. Watanabe, P. M. Watkins, A. T. Watson, I. J. Watson, M. F. Watson, G. Watts, S. Watts, A. T. Waugh, B. M. Waugh, M. S. Weber, P. Weber, A. R. Weidberg, P. Weigell, J. Weingarten, C. Weiser, H. Wellenstein, P. S. Wells, T. Wenaus, D. Wendland, Z. Weng, T. Wengler, S. Wenig, N. Wermes, M. Werner, P. Werner, M. Werth, M. Wessels, J. Wetter, C. Weydert, K. Whalen, S. J. Wheeler-Ellis, A. White, M. J. White, S. White, S. R. Whitehead, D. Whiteson, D. Whittington, F. Wicek, D. Wicke, F. J. Wickens, W. Wiedenmann, M. Wielers, P. Wienemann, C. Wiglesworth, L. A. M. Wiik-Fuchs, P. A. Wijeratne, A. Wildauer, M. A. Wildt, I. Wilhelm, H. G. Wilkens, J. Z. Will, E. Williams, H. H. Williams, W. Willis, S. Willocq, J. A. Wilson, M. G. Wilson, A. Wilson, I. Wingerter-Seez, S. Winkelmann, F. Winklmeier, M. Wittgen, S. J. Wollstadt, M. W. Wolter, H. Wolters, W. C. Wong, G. Wooden, B. K. Wosiek, J. Wotschack, M. J. Woudstra, K. W. Wozniak, K. Wraight, M. Wright, B. Wrona, S. L. Wu, X. Wu, Y. Wu, E. Wulf, B. M. Wynne, S. Xella, M. Xiao, S. Xie, C. Xu, D. Xu, B. Yabsley, S. Yacoob, M. Yamada, H. Yamaguchi, A. Yamamoto, K. Yamamoto, S. Yamamoto, T. Yamamura, T. Yamanaka, J. Yamaoka, T. Yamazaki, Y. Yamazaki, Z. Yan, H. Yang, U. K. Yang, Y. Yang, Z. Yang, S. Yanush, L. Yao, Y. Yao, Y. Yasu, G. V. Ybeles Smit, J. Ye, S. Ye, M. Yilmaz, R. Yoosoofmiya, K. Yorita, R. Yoshida, C. Young, C. J. Young, S. Youssef, D. Yu, J. Yu, J. Yu, L. Yuan, A. Yurkewicz, B. Zabinski, R. Zaidan, A. M. Zaitsev, Z. Zajacova, L. Zanello, A. Zaytsev, C. Zeitnitz, M. Zeman, A. Zemla, C. Zendler, O. Zenin, T. Ženiš, Z. Zinonos, S. Zenz, D. Zerwas, G. Zevi della Porta, Z. Zhan, D. Zhang, H. Zhang, J. Zhang, X. Zhang, Z. Zhang, L. Zhao, T. Zhao, Z. Zhao, A. Zhemchugov, J. Zhong, B. Zhou, N. Zhou, Y. Zhou, C. G. Zhu, H. Zhu, J. Zhu, Y. Zhu, X. Zhuang, V. Zhuravlov, D. Zieminska, N. I. Zimin, R. Zimmermann, S. Zimmermann, S. Zimmermann, M. Ziolkowski, R. Zitoun, L. Živković, V. V. Zmouchko, G. Zobernig, A. Zoccoli, M. zur Nedden, V. Zutshi, L. Zwalinski

**Affiliations:** 1 CERN, 1211 Geneva 23, Switzerland; 2 Physics Department, SUNY Albany, Albany, NY United States of America; 3 Department of Physics, University of Alberta, Edmonton, AB Canada; 4 Department of Physics, Ankara University, Ankara, Turkey; 5 Department of Physics, Dumlupinar University, Kutahya, Turkey; 6 Department of Physics, Gazi University, Ankara, Turkey; 7 Division of Physics, TOBB University of Economics and Technology, Ankara, Turkey; 8 Turkish Atomic Energy Authority, Ankara, Turkey; 9 LAPP, CNRS/IN2P3 and Université de Savoie, Annecy-le-Vieux, France; 10 High Energy Physics Division, Argonne National Laboratory, Argonne, IL United States of America; 11 Department of Physics, University of Arizona, Tucson, AZ United States of America; 12 Department of Physics, The University of Texas at Arlington, Arlington, TX United States of America; 13 Physics Department, University of Athens, Athens, Greece; 14 Physics Department, National Technical University of Athens, Zografou, Greece; 15 Institute of Physics, Azerbaijan Academy of Sciences, Baku, Azerbaijan; 16 Institut de Física d’Altes Energies and Departament de Física de la Universitat Autònoma de Barcelona and ICREA, Barcelona, Spain; 17 Institute of Physics, University of Belgrade, Belgrade, Serbia; 18 Vinca Institute of Nuclear Sciences, University of Belgrade, Belgrade, Serbia; 19 Department for Physics and Technology, University of Bergen, Bergen, Norway; 20 Physics Division, Lawrence Berkeley National Laboratory and University of California, Berkeley, CA United States of America; 21 Department of Physics, Humboldt University, Berlin, Germany; 22 Albert Einstein Center for Fundamental Physics and Laboratory for High Energy Physics, University of Bern, Bern, Switzerland; 23 School of Physics and Astronomy, University of Birmingham, Birmingham, United Kingdom; 24 Department of Physics, Bogazici University, Istanbul, Turkey; 25 Division of Physics, Dogus University, Istanbul, Turkey; 26 Department of Physics Engineering, Gaziantep University, Gaziantep, Turkey; 27 Department of Physics, Istanbul Technical University, Istanbul, Turkey; 28 INFN Sezione di Bologna, Bologna, Italy; 29 Dipartimento di Fisica, Università di Bologna, Bologna, Italy; 30 Physikalisches Institut, University of Bonn, Bonn, Germany; 31 Department of Physics, Boston University, Boston, MA United States of America; 32 Department of Physics, Brandeis University, Waltham, MA United States of America; 33 Universidade Federal do Rio De Janeiro COPPE/EE/IF, Rio de Janeiro, Brazil; 34 Federal University of Juiz de Fora (UFJF), Juiz de Fora, Brazil; 35 Federal University of Sao Joao del Rei (UFSJ), Sao Joao del Rei, Brazil; 36 Instituto de Fisica, Universidade de Sao Paulo, Sao Paulo, Brazil; 37 Physics Department, Brookhaven National Laboratory, Upton, NY United States of America; 38 National Institute of Physics and Nuclear Engineering, Bucharest, Romania; 39 University Politehnica Bucharest, Bucharest, Romania; 40 West University in Timisoara, Timisoara, Romania; 41 Departamento de Física, Universidad de Buenos Aires, Buenos Aires, Argentina; 42 Cavendish Laboratory, University of Cambridge, Cambridge, United Kingdom; 43 Department of Physics, Carleton University, Ottawa, ON Canada; 44 CERN, Geneva, Switzerland; 45 Enrico Fermi Institute, University of Chicago, Chicago, IL United States of America; 46 Departamento de Física, Pontificia Universidad Católica de Chile, Santiago, Chile; 47 Departamento de Física, Universidad Técnica Federico Santa María, Valparaíso, Chile; 48 Institute of High Energy Physics, Chinese Academy of Sciences, Beijing, China; 49 Department of Modern Physics, University of Science and Technology of China, Anhui, China; 50 Department of Physics, Nanjing University, Jiangsu, China; 51 School of Physics, Shandong University, Shandong, China; 52 Laboratoire de Physique Corpusculaire, Clermont Université and Université Blaise Pascal and CNRS/IN2P3, Aubiere Cedex, France; 53 Nevis Laboratory, Columbia University, Irvington, NY United States of America; 54 Niels Bohr Institute, University of Copenhagen, Kobenhavn, Denmark; 55 INFN Gruppo Collegato di Cosenza, Arcavata di Rende, Italy; 56 Dipartimento di Fisica, Università della Calabria, Arcavata di Rende, Italy; 57 Faculty of Physics and Applied Computer Science, AGH University of Science and Technology, Krakow, Poland; 58 The Henryk Niewodniczanski Institute of Nuclear Physics, Polish Academy of Sciences, Krakow, Poland; 59 Physics Department, Southern Methodist University, Dallas, TX United States of America; 60 Physics Department, University of Texas at Dallas, Richardson, TX United States of America; 61 DESY, Hamburg and Zeuthen, Germany; 62 Institut für Experimentelle Physik IV, Technische Universität Dortmund, Dortmund, Germany; 63 Institut für Kern-und Teilchenphysik, Technical University Dresden, Dresden, Germany; 64 Department of Physics, Duke University, Durham, NC United States of America; 65 SUPA - School of Physics and Astronomy, University of Edinburgh, Edinburgh, United Kingdom; 66 INFN Laboratori Nazionali di Frascati, Frascati, Italy; 67 Fakultät für Mathematik und Physik, Albert-Ludwigs-Universität, Freiburg, Germany; 68 Section de Physique, Université de Genève, Geneva, Switzerland; 69 INFN Sezione di Genova, Genova, Italy; 70 Dipartimento di Fisica, Università di Genova, Genova, Italy; 71 E. Andronikashvili Institute of Physics, Tbilisi State University, Tbilisi, Georgia; 72 High Energy Physics Institute, Tbilisi State University, Tbilisi, Georgia; 73 II Physikalisches Institut, Justus-Liebig-Universität Giessen, Giessen, Germany; 74 SUPA - School of Physics and Astronomy, University of Glasgow, Glasgow, United Kingdom; 75 II Physikalisches Institut, Georg-August-Universität, Göttingen, Germany; 76 Laboratoire de Physique Subatomique et de Cosmologie, Université Joseph Fourier and CNRS/IN2P3 and Institut National Polytechnique de Grenoble, Grenoble, France; 77 Department of Physics, Hampton University, Hampton, VA United States of America; 78 Laboratory for Particle Physics and Cosmology, Harvard University, Cambridge, MA United States of America; 79 Kirchhoff-Institut für Physik, Ruprecht-Karls-Universität Heidelberg, Heidelberg, Germany; 80 Physikalisches Institut, Ruprecht-Karls-Universität Heidelberg, Heidelberg, Germany; 81 ZITI Institut für technische Informatik, Ruprecht-Karls-Universität Heidelberg, Mannheim, Germany; 82 Faculty of Applied Information Science, Hiroshima Institute of Technology, Hiroshima, Japan; 83 Department of Physics, Indiana University, Bloomington, IN United States of America; 84 Institut für Astro- und Teilchenphysik, Leopold-Franzens-Universität, Innsbruck, Austria; 85 University of Iowa, Iowa City, IA United States of America; 86 Department of Physics and Astronomy, Iowa State University, Ames, IA United States of America; 87 Joint Institute for Nuclear Research, JINR Dubna, Dubna, Russia; 88 KEK, High Energy Accelerator Research Organization, Tsukuba, Japan; 89 Graduate School of Science, Kobe University, Kobe, Japan; 90 Faculty of Science, Kyoto University, Kyoto, Japan; 91 Kyoto University of Education, Kyoto, Japan; 92 Department of Physics, Kyushu University, Fukuoka, Japan; 93 Instituto de Física La Plata, Universidad Nacional de La Plata and CONICET, La Plata, Argentina; 94 Physics Department, Lancaster University, Lancaster, United Kingdom; 95 INFN Sezione di Lecce, Lecce, Italy; 96 Dipartimento di Matematica e Fisica, Università del Salento, Lecce, Italy; 97 Oliver Lodge Laboratory, University of Liverpool, Liverpool, United Kingdom; 98 Department of Physics, Jožef Stefan Institute and University of Ljubljana, Ljubljana, Slovenia; 99 School of Physics and Astronomy, Queen Mary University of London, London, United Kingdom; 100 Department of Physics, Royal Holloway University of London, Surrey, United Kingdom; 101 Department of Physics and Astronomy, University College London, London, United Kingdom; 102 Laboratoire de Physique Nucléaire et de Hautes Energies, UPMC and Université Paris-Diderot and CNRS/IN2P3, Paris, France; 103 Fysiska institutionen, Lunds universitet, Lund, Sweden; 104 Departamento de Fisica Teorica C-15, Universidad Autonoma de Madrid, Madrid, Spain; 105 Institut für Physik, Universität Mainz, Mainz, Germany; 106 School of Physics and Astronomy, University of Manchester, Manchester, United Kingdom; 107 CPPM, Aix-Marseille Université and CNRS/IN2P3, Marseille, France; 108 Department of Physics, University of Massachusetts, Amherst, MA United States of America; 109 Department of Physics, McGill University, Montreal, QC Canada; 110 School of Physics, University of Melbourne, Victoria, Australia; 111 Department of Physics, The University of Michigan, Ann Arbor, MI United States of America; 112 Department of Physics and Astronomy, Michigan State University, East Lansing, MI United States of America; 113 INFN Sezione di Milano, Milano, Italy; 114 Dipartimento di Fisica, Università di Milano, Milano, Italy; 115 B.I. Stepanov Institute of Physics, National Academy of Sciences of Belarus, Minsk, Republic of Belarus; 116 National Scientific and Educational Centre for Particle and High Energy Physics, Minsk, Republic of Belarus; 117 Department of Physics, Massachusetts Institute of Technology, Cambridge, MA United States of America; 118 Group of Particle Physics, University of Montreal, Montreal, QC Canada; 119 P.N. Lebedev Institute of Physics, Academy of Sciences, Moscow, Russia; 120 Institute for Theoretical and Experimental Physics (ITEP), Moscow, Russia; 121 Moscow Engineering and Physics Institute (MEPhI), Moscow, Russia; 122 Skobeltsyn Institute of Nuclear Physics, Lomonosov Moscow State University, Moscow, Russia; 123 Fakultät für Physik, Ludwig-Maximilians-Universität München, München, Germany; 124 Max-Planck-Institut für Physik (Werner-Heisenberg-Institut), München, Germany; 125 Nagasaki Institute of Applied Science, Nagasaki, Japan; 126 Graduate School of Science and Kobayashi-Maskawa Institute, Nagoya University, Nagoya, Japan; 127 INFN Sezione di Napoli, Napoli, Italy; 128 Dipartimento di Scienze Fisiche, Università di Napoli, Napoli, Italy; 129 Department of Physics and Astronomy, University of New Mexico, Albuquerque, NM United States of America; 130 Institute for Mathematics, Astrophysics and Particle Physics, Radboud University Nijmegen/Nikhef, Nijmegen, Netherlands; 131 Nikhef National Institute for Subatomic Physics and University of Amsterdam, Amsterdam, Netherlands; 132 Department of Physics, Northern Illinois University, DeKalb, IL United States of America; 133 Budker Institute of Nuclear Physics, SB RAS, Novosibirsk, Russia; 134 Department of Physics, New York University, New York, NY United States of America; 135 Ohio State University, Columbus, OH United States of America; 136 Faculty of Science, Okayama University, Okayama, Japan; 137 Homer L. Dodge Department of Physics and Astronomy, University of Oklahoma, Norman, OK United States of America; 138 Department of Physics, Oklahoma State University, Stillwater, OK United States of America; 139 RCPTM, Palacký University, Olomouc, Czech Republic; 140 Center for High Energy Physics, University of Oregon, Eugene, OR United States of America; 141 LAL, Université Paris-Sud and CNRS/IN2P3, Orsay, France; 142 Graduate School of Science, Osaka University, Osaka, Japan; 143 Department of Physics, University of Oslo, Oslo, Norway; 144 Department of Physics, Oxford University, Oxford, United Kingdom; 145 INFN Sezione di Pavia, Pavia, Italy; 146 Dipartimento di Fisica, Università di Pavia, Pavia, Italy; 147 Department of Physics, University of Pennsylvania, Philadelphia, PA United States of America; 148 Petersburg Nuclear Physics Institute, Gatchina, Russia; 149 INFN Sezione di Pisa, Pisa, Italy; 150 Dipartimento di Fisica E. Fermi, Università di Pisa, Pisa, Italy; 151 Department of Physics and Astronomy, University of Pittsburgh, Pittsburgh, PA United States of America; 152 Laboratorio de Instrumentacao e Fisica Experimental de Particulas - LIP, Lisboa, Portugal; 153 Departamento de Fisica Teorica y del Cosmos and CAFPE, Universidad de Granada, Granada, Spain; 154 Institute of Physics, Academy of Sciences of the Czech Republic, Praha, Czech Republic; 155 Faculty of Mathematics and Physics, Charles University in Prague, Praha, Czech Republic; 156 Czech Technical University in Prague, Praha, Czech Republic; 157 State Research Center Institute for High Energy Physics, Protvino, Russia; 158 Particle Physics Department, Rutherford Appleton Laboratory, Didcot, United Kingdom; 159 Physics Department, University of Regina, Regina, SK Canada; 160 Ritsumeikan University, Kusatsu, Shiga Japan; 161 INFN Sezione di Roma I, Roma, Italy; 162 Dipartimento di Fisica, Università La Sapienza, Roma, Italy; 163 INFN Sezione di Roma Tor Vergata, Roma, Italy; 164 Dipartimento di Fisica, Università di Roma Tor Vergata, Roma, Italy; 165 INFN Sezione di Roma Tre, Roma, Italy; 166 Dipartimento di Fisica, Università Roma Tre, Roma, Italy; 167 Faculté des Sciences Ain Chock, Réseau Universitaire de Physique des Hautes Energies - Université Hassan II, Casablanca, Morocco; 168 Centre National de l’Energie des Sciences Techniques Nucleaires, Rabat, Morocco; 169 Faculté des Sciences Semlalia, Université Cadi Ayyad, LPHEA-Marrakech, Marrakech, Morocco; 170 Faculté des Sciences, Université Mohamed Premier and LPTPM, Oujda, Morocco; 171 Faculté des sciences, Université Mohammed V-Agdal, Rabat, Morocco; 172 DSM/IRFU (Institut de Recherches sur les Lois Fondamentales de l’Univers), CEA Saclay (Commissariat a l’Energie Atomique), Gif-sur-Yvette, France; 173 Santa Cruz Institute for Particle Physics, University of California Santa Cruz, Santa Cruz, CA United States of America; 174 Department of Physics, University of Washington, Seattle, WA United States of America; 175 Department of Physics and Astronomy, University of Sheffield, Sheffield, United Kingdom; 176 Department of Physics, Shinshu University, Nagano, Japan; 177 Fachbereich Physik, Universität Siegen, Siegen, Germany; 178 Department of Physics, Simon Fraser University, Burnaby, BC Canada; 179 SLAC National Accelerator Laboratory, Stanford, CA United States of America; 180 Faculty of Mathematics, Physics & Informatics, Comenius University, Bratislava, Slovak Republic; 181 Department of Subnuclear Physics, Institute of Experimental Physics of the Slovak Academy of Sciences, Kosice, Slovak Republic; 182 Department of Physics, University of Johannesburg, Johannesburg, South Africa; 183 School of Physics, University of the Witwatersrand, Johannesburg, South Africa; 184 Department of Physics, Stockholm University, Stockholm, Sweden; 185 The Oskar Klein Centre, Stockholm, Sweden; 186 Physics Department, Royal Institute of Technology, Stockholm, Sweden; 187 Departments of Physics & Astronomy and Chemistry, Stony Brook University, Stony Brook, NY United States of America; 188 Department of Physics and Astronomy, University of Sussex, Brighton, United Kingdom; 189 School of Physics, University of Sydney, Sydney, Australia; 190 Institute of Physics, Academia Sinica, Taipei, Taiwan; 191 Department of Physics, Technion: Israel Institute of Technology, Haifa, Israel; 192 Raymond and Beverly Sackler School of Physics and Astronomy, Tel Aviv University, Tel Aviv, Israel; 193 Department of Physics, Aristotle University of Thessaloniki, Thessaloniki, Greece; 194 International Center for Elementary Particle Physics and Department of Physics, The University of Tokyo, Tokyo, Japan; 195 Graduate School of Science and Technology, Tokyo Metropolitan University, Tokyo, Japan; 196 Department of Physics, Tokyo Institute of Technology, Tokyo, Japan; 197 Department of Physics, University of Toronto, Toronto, ON Canada; 198 TRIUMF, Vancouver, BC Canada; 199 Department of Physics and Astronomy, York University, Toronto, ON Canada; 200 Institute of Pure and Applied Sciences, University of Tsukuba, 1-1-1 Tennodai, Tsukuba, Ibaraki 305-8571 Japan; 201 Science and Technology Center, Tufts University, Medford, MA United States of America; 202 Centro de Investigaciones, Universidad Antonio Narino, Bogota, Colombia; 203 Department of Physics and Astronomy, University of California Irvine, Irvine, CA United States of America; 204 INFN Gruppo Collegato di Udine, Udine, Italy; 205 ICTP, Trieste, Italy; 206 Dipartimento di Chimica, Fisica e Ambiente, Università di Udine, Udine, Italy; 207 Department of Physics, University of Illinois, Urbana, IL United States of America; 208 Department of Physics and Astronomy, University of Uppsala, Uppsala, Sweden; 209 Instituto de Física Corpuscular (IFIC) and Departamento de Física Atómica, Molecular y Nuclear and Departamento de Ingeniería Electrónica and Instituto de Microelectrónica de Barcelona (IMB-CNM), University of Valencia and CSIC, Valencia, Spain; 210 Department of Physics, University of British Columbia, Vancouver, BC Canada; 211 Department of Physics and Astronomy, University of Victoria, Victoria, BC Canada; 212 Department of Physics, University of Warwick, Coventry, United Kingdom; 213 Waseda University, Tokyo, Japan; 214 Department of Particle Physics, The Weizmann Institute of Science, Rehovot, Israel; 215 Department of Physics, University of Wisconsin, Madison, WI United States of America; 216 Fakultät für Physik und Astronomie, Julius-Maximilians-Universität, Würzburg, Germany; 217 Fachbereich C Physik, Bergische Universität Wuppertal, Wuppertal, Germany; 218 Department of Physics, Yale University, New Haven, CT United States of America; 219 Yerevan Physics Institute, Yerevan, Armenia; 220 Domaine scientifique de la Doua, Centre de Calcul CNRS/IN2P3, Villeurbanne Cedex, France

## Abstract

The measurement of the jet energy resolution is presented using data recorded with the ATLAS detector in proton-proton collisions at $\sqrt{s}=7\mbox{ TeV}$. The sample corresponds to an integrated luminosity of 35 pb^−1^. Jets are reconstructed from energy deposits measured by the calorimeters and calibrated using different jet calibration schemes. The jet energy resolution is measured with two different in situ methods which are found to be in agreement within uncertainties. The total uncertainties on these measurements range from 20 % to 10 % for jets within |*y*|<2.8 and with transverse momenta increasing from 30 GeV to 500 GeV. Overall, the Monte Carlo simulation of the jet energy resolution agrees with the data within 10 %.

## Introduction

Precise knowledge of the jet energy resolution is of key importance for the measurement of the cross-sections of inclusive jets, dijets, multijets or vector bosons accompanied by jets [1–4], top-quark cross-sections and mass measurements [5], and searches involving resonances decaying to jets [6, 7]. The jet energy resolution also has a direct impact on the determination of the missing transverse energy, which plays an important role in many searches for new physics with jets in the final state [8, 9]. This article presents the determination with the ATLAS detector [10, 11] of the jet energy resolution in proton-proton collisions at a centre-of-mass energy of $\sqrt{s}=7\mbox{ TeV}$. The data sample was collected during 2010 and corresponds to 35 pb^−1^ of integrated luminosity delivered by the Large Hadron Collider (LHC) [12] at CERN.

The jet energy resolution is determined by exploiting the transverse momentum balance in events containing jets with large transverse momenta (*p*_T_). This article is structured as follows: Sect. 2 describes the ATLAS detector. Sections 3, 4 and 5 respectively introduce the Monte Carlo simulation, the event and jet selection criteria, and the jet calibration methods. The two techniques to estimate the jet energy resolution from calorimeter observables, the *dijet balance method* [13] and the *bisector method* [14], are discussed respectively in Sects. 6 and 7. These methods rely on somewhat different assumptions, which can be validated in data and are sensitive to different sources of systematic uncertainty. As such, the use of these two independent in situ measurements of the jet energy resolution is important to validate the Monte Carlo simulation. Section 8 presents the results obtained for data and simulation for the default jet energy calibration scheme implemented in ATLAS. Section 9 compares the resolutions obtained by applying the two in situ methods to the Monte Carlo simulation and the resolutions determined by comparing the jet energy at calorimeter and particle level. This comparison will be referred to as a closure test. Sources of systematic uncertainty on the jet energy resolution estimated using the available Monte Carlo simulations and collision data are discussed in Sect. 10. The results for other jet energy calibration schemes are discussed in Sects. 11 and 12, and the conclusions can be found in Sect. 13.

## The ATLAS detector

The ATLAS detector is a multi-purpose detector designed to observe particles produced in high energy proton-proton collisions. A detailed description can be found in Refs. [10, 11]. The Inner (tracking) Detector has complete azimuthal coverage and spans the pseudorapidity region |*η*|<2.5.^1^ The Inner Detector consists of layers of silicon pixel, silicon microstrip and transition radiation tracking detectors. These sub-detectors are surrounded by a superconducting solenoid that produces a uniform 2 T axial magnetic field.

The calorimeter system is composed of several sub-detectors. A high-granularity liquid-argon (LAr) electromagnetic sampling calorimeter covers the |*η*|<3.2 range, and it is split into a barrel (|*η*|<1.475) and two end-caps (1.375<|*η*|<3.2). Lead absorber plates are used over its full coverage. The hadronic calorimetry in the barrel is provided by a sampling calorimeter using steel as the absorber material and scintillating tiles as active material in the range |*η*|<1.7. This tile hadronic calorimeter (Tilecal) is separated into a large barrel (|*η*|<0.8) and two smaller extended barrel cylinders, one on either side of the central barrel. In the end-caps, copper/LAr technology is used for the hadronic end-cap calorimeters (HEC), covering the range 1.5<|*η*|<3.2. The copper-tungsten/LAr forward calorimeters (FCal) provide both electromagnetic and hadronic energy measurements, extending the coverage to |*η*|=4.9.

The trigger system consists of a hardware-based Level 1 (L1) and a two-tier, software-based High Level Trigger (HLT). The L1 jet trigger uses a sliding window algorithm with coarse-granularity calorimeter towers. This is then refined using jets reconstructed from calorimeter cells in the HLT.

## Monte Carlo simulation

### Event generators

Data are compared to Monte Carlo (MC) simulations of jets with large transverse momentum produced via strong interactions described by Quantum Chromodynamics (QCD) in proton-proton collisions at a centre-of-mass energy of $\sqrt{s}$ = 7 TeV. The jet energy resolution is derived for several simulation models in order to study its dependence on the event generator, on the parton showering and hadronisation models, and on tunes of other soft model parameters, such as those of the underlying event. The event generators used for this analysis are described below.

1. Pythia 6.4 MC10 tune: The event generator Pythia [15] simulates non-diffractive proton-proton collisions using a 2→2 matrix element at the leading order (LO) of the strong coupling constant to model the hard sub-process, and uses *p*_T_-ordered parton showers to model additional radiation in the leading-logarithm approximation [16]. Multiple parton interactions [17], as well as fragmentation and hadronization based on the Lund string model [18] are also simulated. The parton distribution function (PDF) set used is the modified leading-order MRST LO* set [19]. The parameters used to describe multiple parton interactions are denoted as the ATLAS MC10 tune [20]. This generator and tune are chosen as the baseline for the jet energy resolution studies.
2. The Pythia Perugia2010 tune is an independent tune of Pythia to hadron collider data with increased final-state radiation to better reproduce the jet and hadronic event shapes observed in LEP and Tevatron data [21]. Parameters sensitive to the production of particles with strangeness and related to jet fragmentation have also been adjusted. It is the tune favoured by ATLAS jet shape measurements [22].
3. The Pythia PARP90 modification is an independent systematic variation of Pythia. The variation has been carried out by changing the PARP(90) parameter that controls the energy dependence of the cut-off, deciding whether the events are generated with the matrix element and parton-shower approach, or the soft underlying event [23].
4. Pythia8 [24] is based on the event generator Pythia and contains several modelling improvements, such as fully interleaved *p*_T_-ordered evolution of multiparton interactions and initial- and final-state radiation, and a richer mix of underlying-event processes.
5. The Herwig++ generator [25–28] uses a leading order 2→2 matrix element with angular-ordered parton showers in the leading-logarithm approximation. Hadronization is performed in the cluster model [29]. The underlying event and soft inclusive interactions use hard and soft multiple partonic interaction models [30]. The MRST LO* PDFs [19] are used.
6. Alpgen is a tree-level matrix element generator for hard multi-parton processes (2→*n*) in hadronic collisions [31]. It is interfaced to Herwig to produce parton showers in leading-logarithm approximation, which are matched to the matrix element partons with the MLM matching scheme [32]. Herwig is used for hadronization and Jimmy [33] is used to model soft multiple parton interactions. The LO CTEQ6L1 PDFs [34] are used.

### Simulation of the ATLAS detector

Detector simulation is performed with the ATLAS simulation framework [35] based on Geant4 [36], which includes a detailed description of the geometry and the material of the detector. The set of processes that describe hadronic interactions in the Geant4 detector simulation are outlined in Refs. [37, 38]. The energy deposited by particles in the active detector material is converted into detector signals to mimic the detector read-out. Finally, the Monte Carlo generated events are processed through the trigger simulation of the experiment and are reconstructed and analysed with the same software that is used for data.

### Simulated pile-up samples

The nominal MC simulation does not include additional proton-proton interactions (pile-up). In order to study its effect on the jet energy resolution, two additional MC samples are used. The first one simulates additional proton-proton interactions in the same bunch crossing (in-time pile-up) while the second sample in addition simulates effects on calorimeter cell energies from close-by bunches (out-of-time pile-up). The average number of interactions per event is 1.7 (1.9) for the in-time (in-time plus out-of-time) pile-up samples, which is a good representation of the 2010 data.

## Event and jet selection

The status of each sub-detector and trigger, as well as reconstructed physics objects in ATLAS is continuously assessed by inspection of a standard set of distributions, and data-quality flags are recorded in a database for each luminosity block (of about two minutes of data-taking). This analysis selects events satisfying data-quality criteria for the Inner Detector and the calorimeters, and for track, jet, and missing transverse energy reconstruction [39].

For each event, the reconstructed primary vertex position is required to be consistent with the beamspot, both transversely and longitudinally, and to be reconstructed from at least five tracks with transverse momentum $p_{\mathrm{T}} ^{\mathrm{track}} > 150~\mbox{MeV}$ associated with it. The primary vertex is defined as the one with the highest associated sum of squared track transverse momenta $\varSigma( p_{\mathrm{T}} ^{\mathrm{track}})^{2}$, where the sum runs over all tracks used in the vertex fit. Events are selected by requiring a specific OR combination of inclusive single-jet and dijet calorimeter-based triggers [40, 41]. The combinations are chosen such that the trigger efficiency for each *p*_T_ bin is greater than 99 %. For the lowest *p*_T_ bin (30–40 GeV), this requirement is relaxed, allowing the lowest-threshold calorimeter inclusive single-jet trigger to be used with an efficiency above 95 %.

Jets are reconstructed with the anti-*k*_*t*_ jet algorithm [42] using the FastJet software [43] with radius parameters *R*=0.4 or *R*=0.6, a four-momentum recombination scheme, and three-dimensional calorimeter topological clusters [44] as inputs. Topological clusters are built from calorimeter cells with a signal at least four times higher than the root-mean-square (RMS) of the noise distribution (seed cells). Cells neighbouring the seed which have a signal to RMS-noise ratio ≥2 are then iteratively added. Finally, all nearest neighbour cells are added to the cluster without any threshold.

Jets from non-collision backgrounds (e.g. beam-gas events) and instrumental noise are removed using the selection criteria outlined in Ref. [39].

Jets are categorized according to their reconstructed rapidity in four different regions to account for the differently instrumented parts of the calorimeter:

- Central region (|*y*|<0.8).
- Extended Tile Barrel (0.8≤|*y*|<1.2).
- Transition region (1.2≤|*y*|<2.1).
- End-Cap region (2.1≤|*y*|<2.8).

Events are selected only if the transverse momenta of the two leading jets are above a jet reconstruction threshold of 7 GeV at the electromagnetic scale (see Sect. 5) and within |*y*|≤2.8, at least one of them being in the central region. The analysis is restricted to |*y*|≤2.8 because of the limited number of jets at higher rapidities.

Monte Carlo simulated “particle jets” are defined as those built using the same jet algorithm as described above, but using instead as inputs the stable particles from the event generator (with a lifetime longer than 10 ps), excluding muons and neutrinos.

## Jet energy calibration

Calorimeter jets are reconstructed from calorimeter energy deposits measured at the electromagnetic scale (EM-scale), the baseline signal scale for the energy deposited by electromagnetic showers in the calorimeter. Their transverse momentum is referred to as $p_{\mathrm{T}} ^{\text{EM-scale}}$. For hadrons this leads to a jet energy measurement that is typically 15–55 % lower than the true energy, due mainly to the non-compensating nature of the ATLAS calorimeter [45]. Fluctuations of the hadronic shower, in particular of its electromagnetic content, as well as energy losses in the dead material lead to a degraded resolution and jet energy response compared to particles interacting only electromagnetically. The jet response is defined as the ratio of calorimeter jet *p*_T_ and particle jet *p*_T_ (see Sect. 4), reconstructed with the same algorithm, and matched in *η*−*ϕ* space (see Sect. 9). Several complementary jet calibration schemes with different levels of complexity and different sensitivity to systematic effects have been developed to understand the jet energy measurements. The jet calibration is performed by applying corrections derived from Monte Carlo simulations to restore the jet response to unity. This is referred to as determining the jet energy scale (JES).

The analysis presented in this article aims to determine the jet energy resolution for jets reconstructed using various JES strategies. A simple calibration, referred to as the EM+JES calibration scheme, has been chosen for the first physics analysis of the 2010 data [39]. It allows a direct evaluation of the systematic uncertainties from single-hadron response measurements and is therefore suitable for first physics analyses. More sophisticated calibration techniques to improve the jet resolution and reduce partonic flavour response differences have also been developed. They are the Local Cluster Weighting (LCW), the Global Cell Weighting (GCW) and the Global Sequential (GS) methods [39]. In addition to these calorimeter calibration schemes, a Track-Based Jet Correction (TBJC) has been derived to adjust the response and reduce fluctuations on a jet-by-jet basis without changing the average jet energy scale. These calibration techniques are briefly described below.

### The EM+JES calibration

For the analysis of the first proton-proton collisions, a simple Monte Carlo simulation-based correction is applied as the default to restore the hadronic energy scale on average. The EM+JES calibration scheme applies corrections as a function of the jet transverse momentum and pseudorapidity to jets reconstructed at the electromagnetic scale. The main advantage of this approach is that it allows the most direct evaluation of the systematic uncertainties. The uncertainty on the absolute jet energy scale was determined to be less than ±2.5 % in the central calorimeter region (|*y*|<0.8) and ±14 % in the most forward region (3.2≤|*y*|<4.5) for jets with *p*_T_>30 GeV [39]. These uncertainties were evaluated using test-beam results, single hadron response in situ measurements, comparison with jets built from tracks, *p*_T_ balance in dijet and *γ*+jet events, estimations of pile-up energy deposits, and detailed Monte Carlo comparisons.

### The Local Cluster Weighting (LCW) calibration

The LCW calibration scheme uses properties of clusters to calibrate them individually *prior* to jet finding and reconstruction. The calibration weights are determined from Monte Carlo simulations of charged and neutral pions according to the cluster topology measured in the calorimeter. The cluster properties used are the energy density in the cells forming them, the fraction of their energy deposited in the different calorimeter layers, the cluster isolation and its depth in the calorimeter. Corrections are applied to the cluster energy to account for the energy deposited in the calorimeter but outside of clusters and energy deposited in material before and in between the calorimeters. Jets are formed from calibrated clusters. A final jet-level energy correction based on the same procedure as for the EM+JES case is applied to attain unity response, but with corrections that are numerically smaller. The resulting jet energy calibration is denoted as LCW+JES.

### The Global Cell Weighting (GCW) calibration

The GCW calibration scheme attempts to compensate for the different calorimeter response to hadronic and electromagnetic energy deposits at cell level. The hadronic signal is characterized by low cell energy densities and, thus, a positive weight is applied. The weights, which depend on the cell energy density and the calorimeter layer only, are determined by minimizing the jet resolution evaluated by comparing reconstructed and particle jets in Monte Carlo simulation. They correct for several effects at once (calorimeter non-compensation, dead material, etc.). A jet-level correction is applied to jets reconstructed from weighted cells to account for global effects. The resulting jet energy calibration is denoted as GCW+JES.

### The Global Sequential (GS) calibration

The GS calibration scheme uses the longitudinal and transverse structure of the jet calorimeter shower to compensate for fluctuations in the jet energy measurement. In this scheme the jet energy response is first calibrated with the EM+JES calibration. Subsequently, the jet properties are used to exploit the topology of the energy deposits in the calorimeter to characterize fluctuations in the hadronic shower development. These corrections are applied such that the mean jet energy is left unchanged, and each correction is applied sequentially. This calibration is designed to improve the jet energy resolution without changing the average jet energy scale.

### Track-based correction to the jet calibration

Regardless of the inputs, algorithms and calibration methods chosen for calorimeter jets, more information on the jet topology can be obtained from reconstructed tracks associated to the jet. Calibrated jets have an average energy response close to unity. However, the energy of an individual jet can be over- or underestimated depending on several factors, for example: the ratio of the electromagnetic and hadronic components of the jet; the fraction of energy lost in dead material, in either the inner detector, the solenoid, the cryostat before the LAr, or the cryostat between the LAr and the TileCal. The reconstructed tracks associated to the jet are sensitive to some of these effects and therefore can be used to correct the calibration on a jet-by-jet basis.

In the method referred to as Track-Based Jet Correction (TBJC) [45], the response is adjusted depending on the number of tracks associated with the jet. The jet energy response is observed to decrease with increasing track multiplicity of the jets, mainly because the ratio of the electromagnetic to the hadronic component decreases on average as the number of tracks increases. In effect, a low charged-track multiplicity typically indicates a predominance of neutral hadrons, in particular *π* ^0^s which yield electromagnetic deposits in the calorimeter with *R*≃1. A large number of charged particles, on the contrary, signals a more dominant hadronic component, with a lower response due to the non-compensating nature of the calorimeter (*h*/*e*<1). The TBJC method is designed to be applied as an option in addition to any JES calibration scheme, since it does not change the average response, to reduce the jet-to-jet energy fluctuations and improve the resolution.

## In situ jet resolution measurement using the dijet balance method

Two methods are used in dijet events to measure in situ the fractional jet *p*_T_ resolution, *σ*(*p*_T_)/*p*_T_, which at fixed rapidity is equivalent to the fractional jet energy resolution, *σ*(*E*)/*E*. The first method, presented in this section, relies on the approximate scalar balance between the transverse momenta of the two leading jets and measures the sensitivity of this balance to the presence of extra jets directly from data. The second one, presented in the next section, uses the projection of the vector sum of the leading jets’ transverse momenta on the coordinate system bisector of the azimuthal angle between the transverse momentum vectors of the two jets. It takes advantage of the very different sensitivities of each of these projections to the underlying physics of the dijet system and to the jet energy resolution.

### Measurement of resolution from asymmetry

The dijet balance method for the determination of the jet *p*_T_ resolution is based on momentum conservation in the transverse plane. The asymmetry between the transverse momenta of the two leading jets *A*(*p*_T,1_,*p*_T,2_) is defined as

1 $$ A(p_{{\mathrm{T}},1},p_{{\mathrm{T}},2}) \equiv\frac{p_{{\mathrm{T}},1}-p_{{\mathrm{T}},2}}{p_{{\mathrm{T}} ,1}+p_{{\mathrm{T}},2}}, $$

where *p*_T,1_ and *p*_T,2_ refer to the randomly ordered transverse momenta of the two leading jets. The width *σ*(*A*) of a Gauss distribution fitted to *A*(*p*_T,1_,*p*_T,2_) is used to characterize the asymmetry distribution and determine the jet *p*_T_ resolutions.

For events with exactly two particle jets that satisfy the hypothesis of momentum balance in the transverse plane, and requiring both jets to be in the same rapidity region, the relation between *σ*(*A*) and the fractional jet resolution is given by

2 $$ \sigma(A) \simeq\frac{\sqrt{{\sigma^2(p_{{\mathrm{T}},1}) + \sigma^2(p_{{\mathrm{T}} ,2})}}}{\langle p_{{\mathrm{T}},1} + p_{{\mathrm{T}},2} \rangle} \simeq \frac{1}{\sqrt{2}} \frac{\sigma( p_{\mathrm {T}} )}{ p_{\mathrm{T}} }, $$

where *σ*(*p*_T,1_)=*σ*(*p*_T,2_)=*σ*(*p*_T_), since both jets are in the same *y* region.

If one of the two leading jets (*j*) is in the rapidity bin being probed and the other one (*i*) in a reference *y* region where the resolution may be different, the fractional jet *p*_T_ resolution is given by

3 $$ \frac{\sigma(p_{\mathrm{T}})}{p_{\mathrm{T}}} {\bigg\vert}_{(j)} = \sqrt{4\sigma^{2}(A_{(i,j)}) - 2\sigma^{2}(A_{(i)})} , $$

where *A*_(*i*,*j*)_ is measured in a topology with the two jets in different rapidity regions and where (*i*)≡(*i*,*i*) denotes both jets in the same *y* region.

**Fig. 1:**
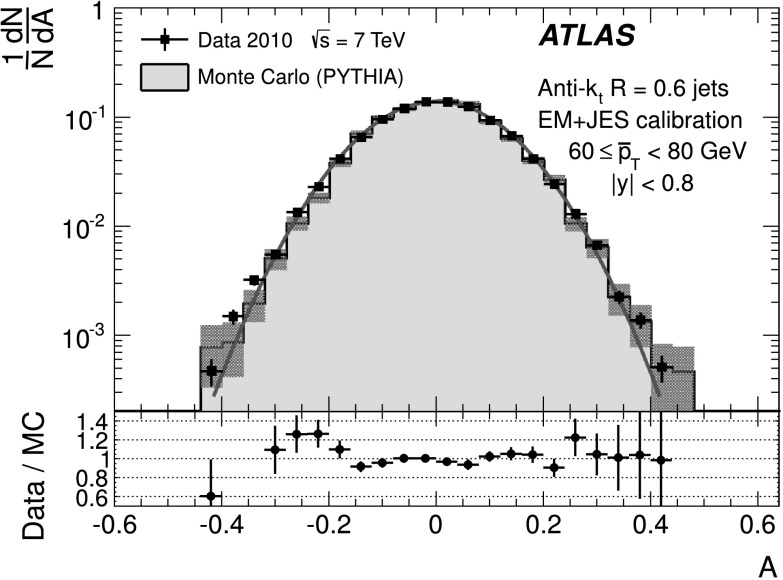
Asymmetry distribution as defined in Eq. (1) for $60\le\bar{p}_{\mathrm{T}}<80~\mbox{GeV}$ and |*y*|<0.8. Data (*points with error bars*) and Monte Carlo simulation (*histogram with shaded error bands*) are overlaid, together with a Gaussian function fit to the data. The *lower panel* shows the ratio between data and MC simulation. The errors shown are only statistical

The back-to-back requirement is approximated by an azimuthal angle cut between the leading jets, Δ*ϕ*(*j*_1_,*j*_2_)≥2.8, and a veto on the third jet momentum, $p^{\text{EM-scale}}_{{\mathrm{T}},3} < 10~\text{GeV}$, with no rapidity restriction. The resulting asymmetry distribution is shown in Fig. 1 for a $\bar{p}_{\mathrm{T}}\equiv(p_{{\mathrm {T}},1} + p_{{\mathrm{T}},2})/2$ bin of $60~\text{GeV}\le\bar{p}_{\mathrm{T}}<80~\text{GeV}$, in the central region (|*y*|<0.8). Reasonable agreement in the bulk is observed between data and Monte Carlo simulation.

### Soft radiation correction

Although requirements on the azimuthal angle between the leading jets and on the third jet transverse momentum are designed to enrich the purity of the back-to-back jet sample, it is important to account for the presence of additional soft particle jets not detected in the calorimeter.

In order to estimate the value of the asymmetry for a pure particle dijet event, $\sigma( p_{\mathrm{T}} )/ p_{\mathrm{T}} \equiv\sqrt{2}\, \sigma(A)$ is recomputed allowing for the presence of an additional third jet in the sample for a series of $p_{{\mathrm{T}},3}^{\text{EM-scale}}$ threshold values up to 20 GeV. The cut on the third jet is placed at the EM-scale to be independent of calibration effects and to have a stable reference for all calibration schemes. For each *p*_T_ bin, the jet energy resolutions obtained with the different $p_{{\mathrm{T}} ,3}^{\text{EM-scale}}$ cuts are fitted with a straight line and extrapolated to $p_{{\mathrm{T}},3}^{\text{EM-scale}}\rightarrow0$, in order to estimate the expected resolution for an ideal dijet topology

$$ \frac{\sigma(p_{\mathrm{T}})}{p_{\mathrm{T}}}\bigg|_{p_{{\mathrm{T}},3}^{\text{EM-scale}} \rightarrow 0}. $$

**Fig. 2:**
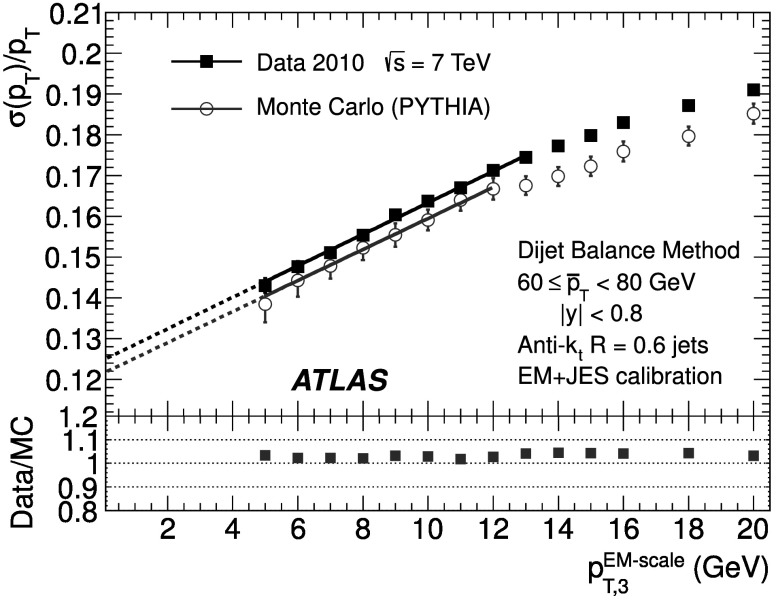
Fractional jet *p*_T_ resolutions, from Eq. (2), measured in events with $60 \le\bar{p}_{\mathrm{T}} <80~\mbox{GeV}$ and with third jet with *p*_T_ less than $p_{{\mathrm{T}},3}^{\text{EM-scale}}$, as a function of $p_{{\mathrm{T}},3}^{\text{EM-scale}}$, for data (*squares*) and Monte Carlo simulation (*circles*). The *solid lines* correspond to linear fits while the *dashed lines* show the extrapolations to $p_{{\mathrm{T}},3}^{\text{EM-scale}}= 0$. The *lower panel* shows the ratio between data and MC simulation. The errors shown are only statistical

The dependence of the jet *p*_T_ resolution on the presence of a third jet is illustrated in Fig. 2. The linear fits and their extrapolations for a $\bar{p}_{\mathrm{T}}$ bin of $60 \le\bar{p}_{\mathrm{T}}<80~\mbox{GeV}$ are shown. Note that the resolutions become systematically broader as the $p_{{\mathrm{T}},3}^{\text{EM-scale}}$ cut increases. This is a clear indication that the jet resolution determined from two-jet topologies depends on the presence of additional radiation and on the underlying event.

A soft radiation (SR) correction factor, $K_{\mathrm{soft}}(\bar {p}_{\mathrm{T}} )$, is obtained from the ratio of the values of the linear fit at 0 GeV and at 10 GeV:

4 $$ K_{\mathrm{soft}}(\bar{p}_{\mathrm{T}}) = \frac{\frac{\sigma( p_{\mathrm{T}} )}{ p_{\mathrm{T}} }|_{p_{{\mathrm{T}},3}^{\text{EM-scale}} \longrightarrow 0~\text{GeV}}}{ \frac{\sigma( p_{\mathrm{T}} )}{ p_{\mathrm{T}} }|_{p_{{\mathrm{T}},3}^{\text{EM-scale}}=10~\text{GeV}}} . $$

This multiplicative correction is applied to the resolutions extracted from the dijet asymmetry for $p^{\text{EM-scale}}_{{\mathrm{T}},3}<10~\mbox{GeV}$ events. The correction varies from 25 % for events with $\bar{p}_{\mathrm{T}}$ of 50 GeV down to 5 % for $\bar{p}_{\mathrm{T}}$ of 400 GeV. In order to limit the statistical fluctuations, $K_{\mathrm{soft}}(\bar{p}_{\mathrm{T}})$ is fit with a parameterization of the form $K_{\mathrm{soft}}(\bar{p}_{\mathrm{T}}) = a + b/ (\log\bar {p}_{\mathrm{T}})^{2}$, which was found to describe the distribution well, within uncertainties. The differences in the resolution due to other parameterizations of *K* were studied and treated as a systematic uncertainty, resulting in a relative uncertainty of about 6 % (see Sect. 10).

### Particle balance correction

**Fig. 3:**
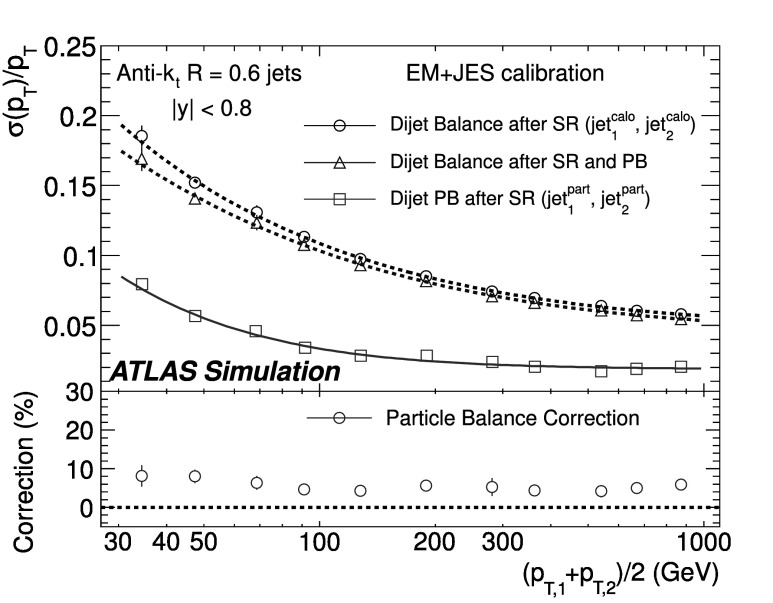
Fractional jet resolution obtained in simulation using the dijet balance method, shown as a function of $\bar{p}_{\mathrm{T}}$, both before (*circles*) and after the particle-balance (PB) correction (*triangles*). Also shown is the dijet PB correction itself (*squares*) and, in the *lower panel*, its relative size with respect to the fractional jet resolution. The *curves* correspond to fits with the functional form in Eq. (9). The errors shown are only statistical

The *p*_T_ difference between the two calorimeter jets is not solely due to resolution effects, but also to the balance between the respective particle jets, 


The measured difference (left side) is decomposed into resolution fluctuations (the first two terms on the right side) plus a particle-level balance (PB) term that originates from out-of-jet showering in the particle jets. In order to correct for this contribution, the particle-level balance is estimated using the same technique (asymmetry plus soft radiation correction) as for calorimeter jets. The contribution of the dijet PB after the SR correction is subtracted in quadrature from the in situ resolution for both data and Monte Carlo simulation. The result of this procedure is shown for simulated events in the central region in Fig. 3. The relative size of the particle-level balance correction with respect to the measured resolutions is of the order of 5 %.

## In situ jet resolution measurement using the bisector method

### Bisector rationale

**Fig. 4:**
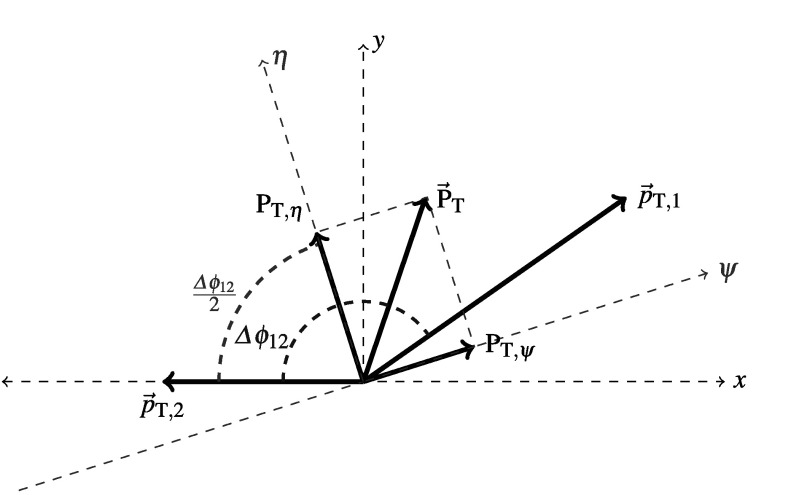
Variables used in the bisector method. The *η*-axis corresponds to the azimuthal angular bisector of the dijet system in the plane transverse to the beam, while the *ψ*-axis is defined as the one orthogonal to the *η*-axis

The bisector method [14] is based on a transverse balance vector, $\vec{\mathrm{P}}_{\mathrm{T}}$, defined as the sum of the momenta of the two leading jets in dijet events, $\vec{p}_{{\mathrm{T}},1}$ and $\vec{p}_{{\mathrm{T}},2}$. This vector is projected along an orthogonal coordinate system in the transverse plane, (*ψ*,*η*), where *η* is chosen in the direction that bisects Δ*ϕ*_12_, the angle formed by $\vec{p}_{{\mathrm{T}},1}$ and $\vec{p}_{{\mathrm{T}},2}$. This is illustrated in Fig. 4.

For a perfectly balanced dijet event, $\vec{\mathrm{P}}_{{\mathrm{T}}}=0$. There are of course a number of sources that give rise to significant fluctuations around this value, and thus to a non-zero variance of its *ψ* and *η* components, denoted $\sigma_{\psi}^{2} $ and $\sigma_{\eta}^{2}$, respectively.

At particle level, $\vec{\mathrm{P}}^{{\mathrm{part}}}_{\mathrm{T}}$ receives contributions mostly from initial-state radiation. This effect is expected to be isotropic in (*ψ*,*η*), leading to similar fluctuations in both components, $\sigma^{{\mathrm{part}}}_{\psi}=\sigma^{{\mathrm{part}}}_{\eta}$. The validity of this assumption, which is at the root of the method, can be studied with Monte Carlo simulations and with data. The precision with which it can be assessed is considered as a systematic uncertainty (see Sect. 7.2).

At calorimeter level, $\vec{\mathrm{P}}^{{\mathrm{calo}}}_{{\mathrm{T}}}$ will further differ from zero due to detector effects. Its *ψ* component, $\mathrm{P}^{\mathrm{calo}}_{{\mathrm{T}} \psi}=p^{\mathrm{calo}}_{{{\mathrm{T}},1}{ \psi}}-p^{\mathrm{calo}}_{{{\mathrm{T}},2}{ \psi}}$, can be decomposed into three contributions,  where the first two terms correspond to fluctuations due to the detector *p*_T_ resolution, and the last one to the particle jet imbalance. Taking the variance of the sum of these three independent terms yields

5 $$ \sigma^{2\ {\mathrm{calo}}}_{\psi}\simeq\sigma^{2\ {\mathrm {part}}}_{\psi}+2 \sigma^2(p_{\mathrm{T}})\bigl\langle\sin^2(\Delta \phi_{12}/2)\bigr\rangle $$

where the following relations have been used  Here *σ*(*p*_T_) corresponds to *σ*(*p*_T,1_)≃*σ*(*p*_T,2_), as both jets have the same *p*_T_ resolution since they belong to the same *y* region. A relation similar to Eq. (5) holds for the *η* component:

6 $$ \sigma^{2\ {\mathrm{calo}}}_{\eta}\simeq\sigma^{2\ {\mathrm {part}}}_{\eta}+2 \sigma^2(p_{\mathrm{T}})\bigl\langle\cos^2(\Delta \phi_{12}/2)\bigr\rangle. $$

Subtracting Eq. (6) from Eq. (5), and using $\sigma^{{\mathrm{part}}}_{\psi}=\sigma^{{\mathrm{part}}}_{\eta }$, yields

7 $$ \frac{\sigma( p_{\mathrm{T}} )}{ p_{\mathrm {T}} } \simeq \frac{\sqrt{\sigma^{2\ {\mathrm{calo}}}_{\psi}-\sigma^{2\ {\mathrm {calo}}}_{\eta}}}{ \sqrt{2} p_{\mathrm{T}} \sqrt{\langle|\cos \Delta\phi_{12}|\rangle}} , $$

where the fractional jet *p*_T_ resolution, *σ*(*p*_T_)/*p*_T_, is expressed in terms of calorimeter observables only. The contribution from soft radiation and the underlying event is minimised by subtracting in quadrature *σ*_*η*_ from *σ*_*ψ*_.

If one of the leading jets (*j*) belongs to the rapidity region being probed, and the other one (*i*) to a previously measured reference *y* region, then

8 $$ \frac{\sigma( p_{\mathrm{T}} )}{ p_{\mathrm {T}} } {\bigg\vert}_{(j)} \simeq \sqrt{ \frac{\sigma^{2\ {\mathrm{calo}}}_{\psi}-\sigma^{2\ {\mathrm {calo}}}_{\eta}}{ p^2_{\mathrm{T}} \langle|\cos\Delta\phi_{12}|\rangle} {\bigg\vert }_{(i,j)} - \frac{\sigma^{2}( p_{\mathrm{T}} )}{ p^2_{\mathrm{T}} } { \bigg\lvert}_{(i)}}. $$

**Fig. 5:**
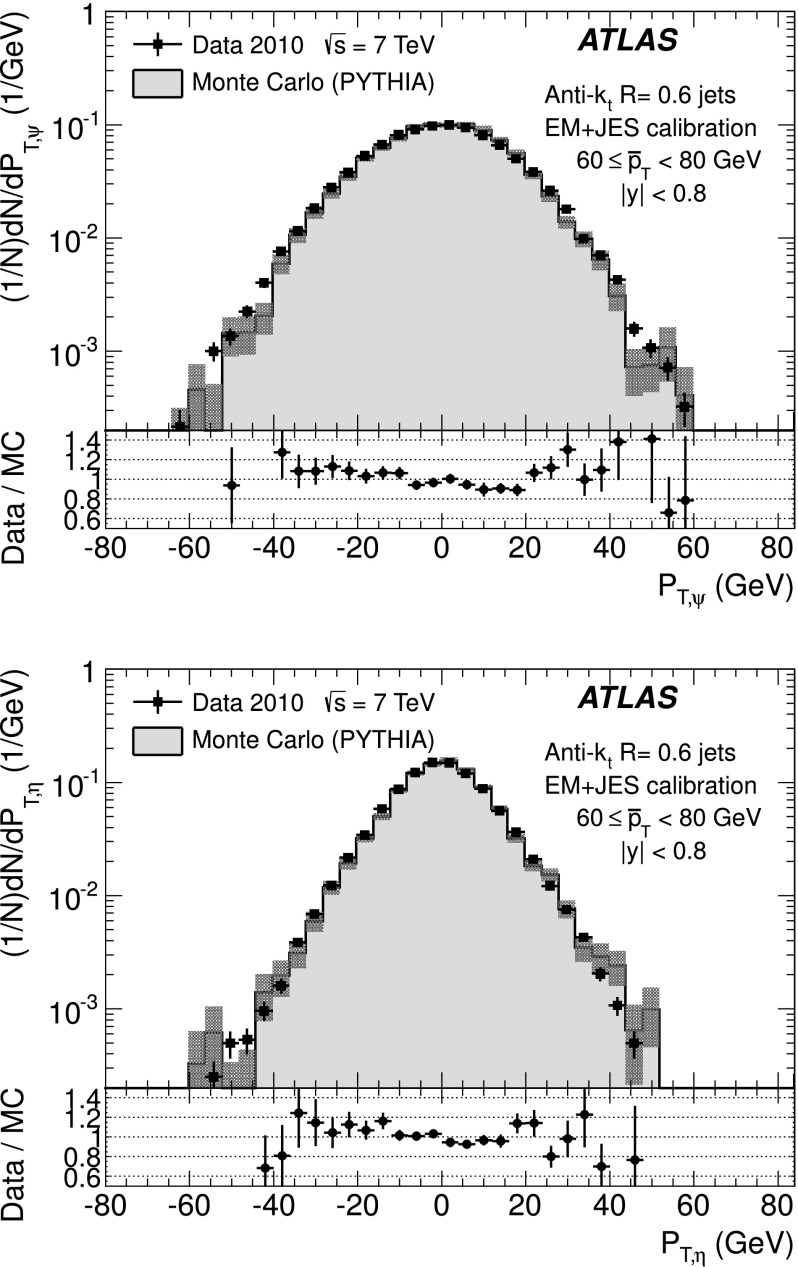
Distributions of the P_T*ψ*_ (*top*) and P_T*η*_ (*bottom*) components of the balance vector $\vec{\mathrm{P}}_{\mathrm{T}}$, for $60\le\bar{p}_{\mathrm {T}}<80~\mbox{GeV}$. The data (*points with error bars*) and Monte Carlo simulation (*histogram with shaded error bands*) are overlaid. The *lower panel* shows the ratio between data and MC simulation. The errors shown are only statistical

**Fig. 6:**
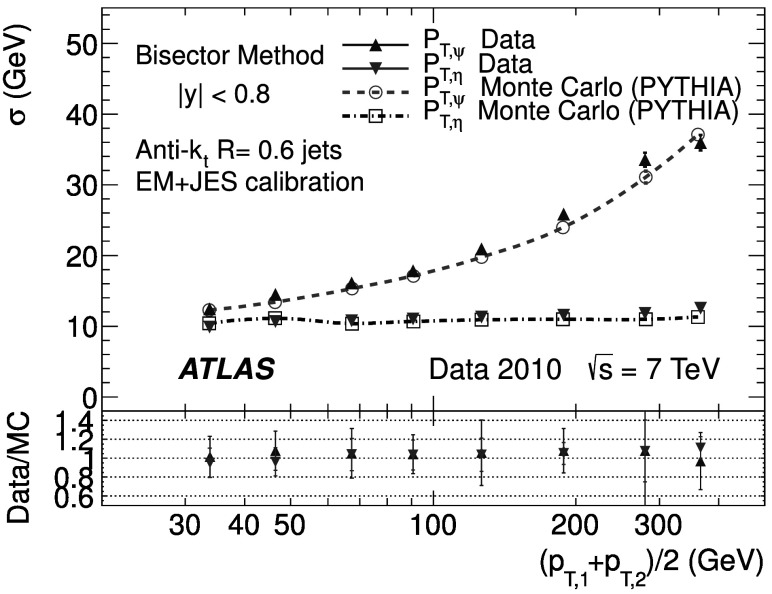
Standard deviations of P_T*ψ*_ and P_T*η*_, the components of the balance vector, as a function of $\bar{p}_{\mathrm{T}}$. MC simulation points are joined by *lines*. The *lower panel* shows the ratio between data and MC simulation. The errors shown are only statistical

The dispersions *σ*_*ψ*_ and *σ*_*η*_ are extracted from Gaussian fits to the P_T*ψ*_ and P_T*η*_ distributions in bins of $\bar {p}_{\mathrm{T}}$. There is no Δ*ϕ* cut imposed between the leading jets, but it is implicitly limited by a $p_{{\mathrm{T}},3}^{\text{EM-scale}}<10~\mbox{GeV}$ requirement on the third jet, as discussed in the next section. Figure 5 compares the distributions of P_T*ψ*_ and P_T*η*_ between data and Monte Carlo simulation in the momentum bin $60\le\bar{p}_{\mathrm{T}}<80~\mbox{GeV}$. The distributions agree within statistical fluctuations. The resolutions obtained from the P_T*ψ*_ and P_T*η*_ components of the balance vector are summarised in the central region as a function of $\bar {p}_{\mathrm{T}}$ in Fig. 6. As expected, the resolution on the *η* component does not vary with the jet *p*_T_, while the resolution on the *ψ* component degrades as the jet *p*_T_ increases.

### Validation of the soft radiation isotropy with data

**Fig. 7:**
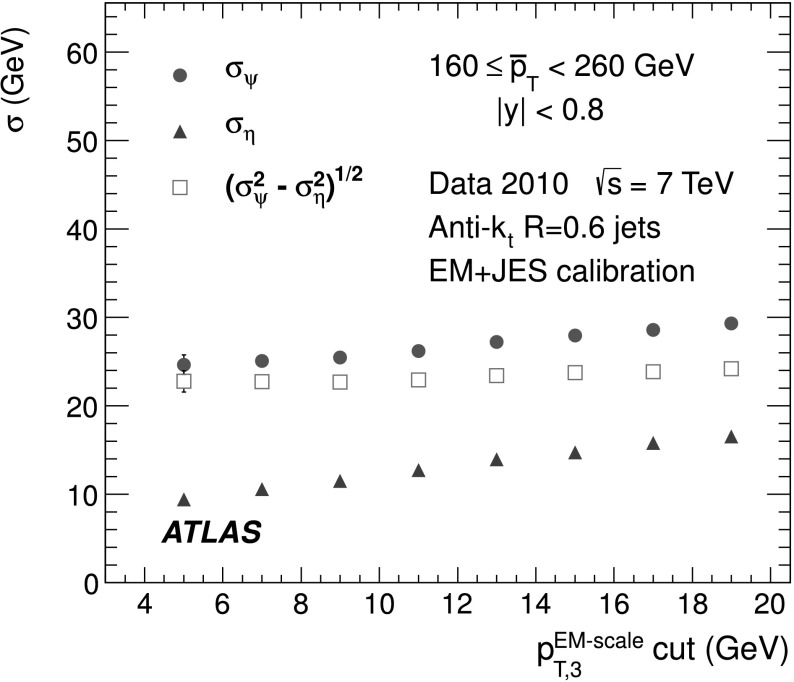
Standard deviations $\sigma^{\mathrm{calo}}_{\psi}$, $\sigma^{\mathrm{calo}}_{\eta}$ and $[(\sigma^{2}_{\psi}-\sigma^{2}_{\eta})^{\mathrm {calo}}]^{1/2}$ as a function of the upper $p_{{\mathrm{T}},3}^{\text{EM-scale}}$ cut, for *R*=0.6 anti-*k*_*t*_ jets with $160\le\bar{p}_{\mathrm{T}}<260~\mbox{GeV}$. The errors shown are only statistical

Figure 7 shows the width of the *ψ* and *η* components of $\vec{\mathrm{P}}_{{\mathrm{T}}}$ as a function of the $p_{{\mathrm{T}} ,3}^{\text{EM-scale}}$ cut, for anti-*k*_*t*_ jets with *R*=0.6. The two leading jets are required to be in the same rapidity region, |*y*|<0.8, while there is no rapidity restriction for the third jet. As expected, both components increase due to the contribution from soft radiation as the *p*_T,3_ cut is increased. Also shown as a function of the $p_{{\mathrm{T}},3}^{\text{EM-scale}}$ cut is the square-root of the difference between their variances, which yields the fractional momentum resolution when divided by $2\langle p^{2}_{\mathrm{T}} \rangle\langle \cos\Delta\phi\rangle$.

It is observed in Fig. 7 that the difference $(\sigma^{2}_{\psi}-\sigma^{2}_{\eta})^{\mathrm{calo}}$ remains almost constant, within statistical uncertainties, up to $p_{{\mathrm{T}},3}^{\text{EM-scale}}\simeq$ 20 GeV for $160\leq\bar{p}_{\mathrm{T}}<260~\mbox{GeV}$. The same behaviour is observed for other $\bar{p}_{\mathrm{T}}$ ranges. This cancellation demonstrates that the isotropy assumption used for the bisector method is consistent with the data over a wide range of choices of $p_{{\mathrm{T}},3}^{\text{EM-scale}}$ without the need for requiring an explicit Δ*ϕ* cut between the leading jets. The precision with which it can be ascertained that the data is consistent with $\sigma^{\mathrm{part}}_{\psi}=\sigma^{\mathrm {part}}_{\eta}$ is taken conservatively as a systematic uncertainty on the method, of about 4–5 % at 50 GeV (see Sect. 10).

## Performance for the EM+JES calibration

**Fig. 8:**
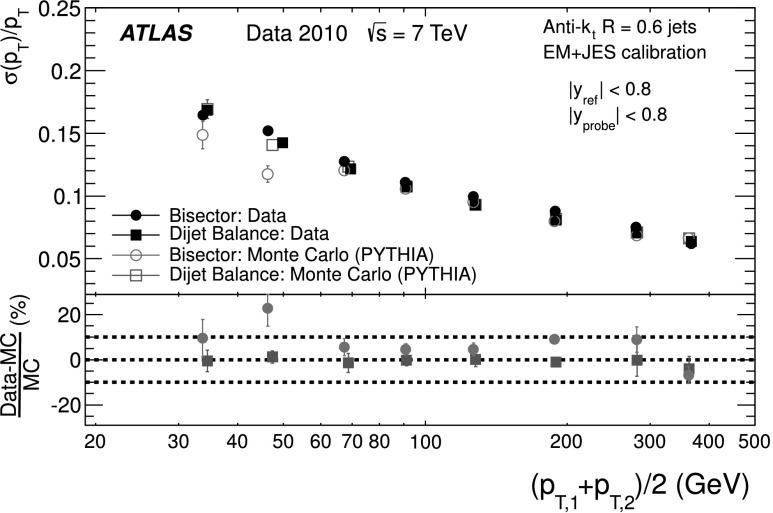
Fractional jet *p*_T_ resolution for the dijet balance and bisector methods as a function of $\bar{p}_{\mathrm{T}}$. The *lower panel* shows the relative difference between data and Monte Carlo results. The *dotted lines* indicate a relative difference of ±10 %. Both methods are found to be in agreement within 10 % between data and Monte Carlo simulation. The errors shown are only statistical

The performances of the dijet balance and bisector methods are compared for both data and Monte Carlo simulation as a function of jet *p*_T_ for jets reconstructed in the central region with the anti-*k*_*t*_ algorithm with *R*=0.6 and using the EM+JES calibration scheme. The results are shown in Fig. 8. The resolutions obtained from the two independent in situ methods are in good agreement with each other within the statistical uncertainties. The agreement between data and Monte Carlo simulation is also good within the statistical precision.

**Fig. 9:**
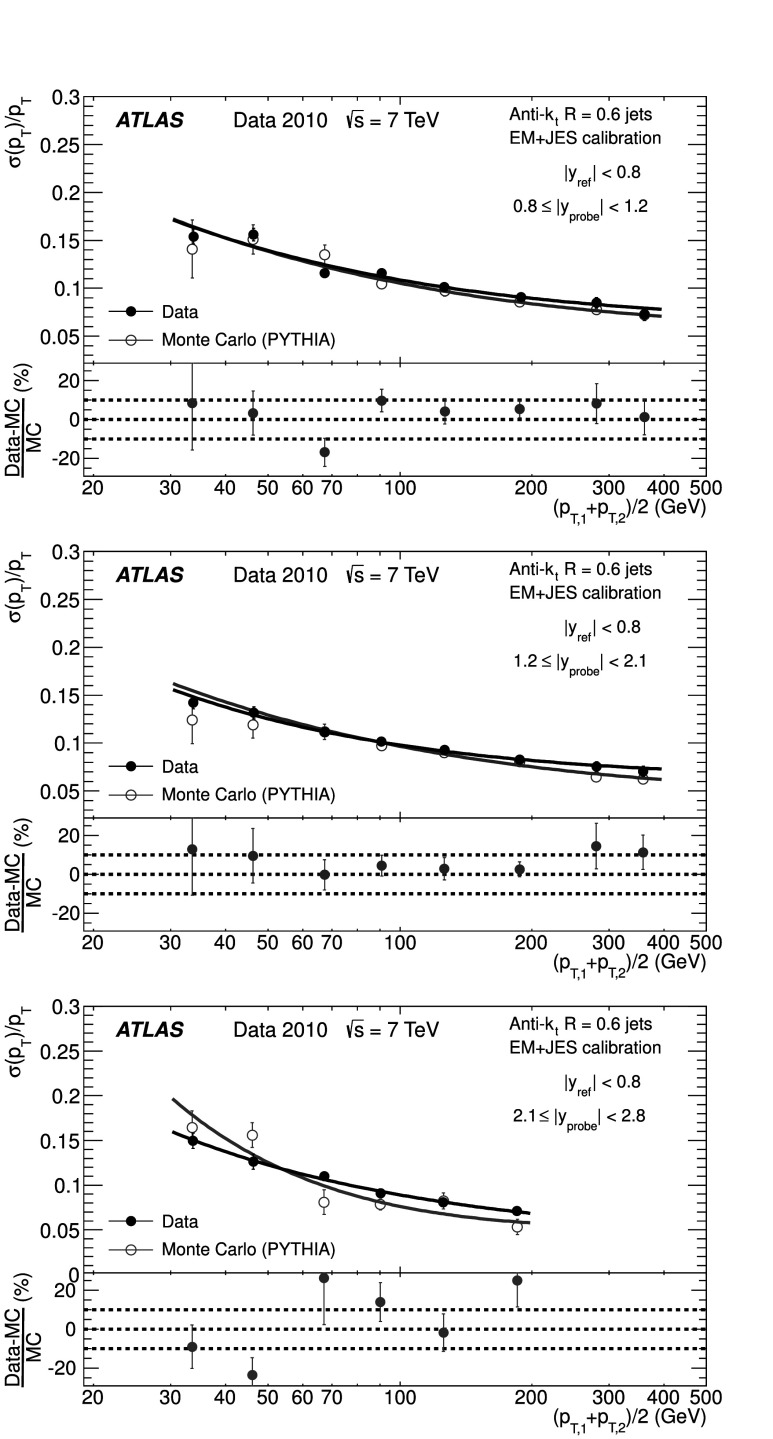
Fractional jet *p*_T_ resolution as a function of $\bar {p}_{\mathrm{T}} $ for anti-*k*_*t*_ with *R*=0.6 jets in the Extended Tile Barrel (*top*), Transition (*center*) and End-Cap (*bottom*) regions using the bisector method. In the *lower panel* of each figure, the relative difference between the data and the MC simulation results is shown. The *dotted lines* indicate a relative difference of ±10 %. The *curves* correspond to fits with the functional form in Eq. (9). The errors shown are only statistical

The resolutions for the three jet rapidity bins with |*y*|>0.8, the Extended Tile Barrel, the Transition and the End-Cap regions, are measured using Eqs. (3) and (8), taking the central region as the reference. The results for the bisector method are shown in Fig. 9. Within statistical errors the resolutions obtained for data and Monte Carlo simulation are in agreement within ±10% over most of the *p*_T_-range in the various regions.

Figure 9 shows that dependences are well described by fits to the standard functional form expected for calorimeter-based resolutions, with three independent contributions, the effective noise (*N*), stochastic (*S*) and constant (*C*) terms.

9 $$ \frac{\sigma( p_{\mathrm{T}} )}{ p_{\mathrm {T}} } = \frac{N}{ p_{\mathrm{T}} }\oplus\frac{S}{\sqrt { p_{\mathrm{T}} }}\oplus C. $$

The *N* term is due to external noise contributions that are not (or only weakly) dependent on the jet *p*_T_, and include the electronics and detector noise, and contributions from pile-up. It is expected to be significant in the low-*p*_T_ region, below ∼30 GeV. The *C* term encompasses the fluctuations that are a constant fraction of the jet *p*_T_, assumed at this early stage of data-taking to be due to real signal lost in passive material (e.g. cryostats and solenoid coil), to non-uniformities of response across the calorimeter, etc. It is expected to dominate the high-*p*_T_ region, above 400 GeV. For intermediate values of the jet *p*_T_, the statistical fluctuations, represented by the *S* term, become the limiting factor of the resolution. With the present data sample that covers a restricted *p*_T_ range, 30 GeV≤*p*_T_<500 GeV, there is a high degree of correlation between the fitted parameters and it is not possible to unequivocally disentangle their contributions.

## Closure test using Monte Carlo simulation

The Monte Carlo simulation expected resolution is derived considering matched particle and calorimeter jets in the event, with no back-to-back geometry requirements. Matching is done in *η*–*ϕ* space, and jets are associated if $\Delta R = \sqrt{(\Delta \eta)^{2} +(\Delta\phi)^{2} }$ <0.3. The jet response is defined as $p_{\mathrm{T}} ^{\mathrm{calo}} / p_{\mathrm {T}} ^{\mathrm{part}}$, in bins of $p_{\mathrm{T}} ^{\mathrm {part}}$, where $p_{\mathrm{T}} ^{\mathrm{calo}}$ and $p_{\mathrm {T}} ^{\mathrm{part}}$ correspond to the transverse momentum of the reconstructed jet and its matched particle jet, respectively. The jet response distribution is modelled by a fitted Gauss distribution, and its standard deviation is defined as the truth jet *p*_T_ resolution.

**Fig. 10:**
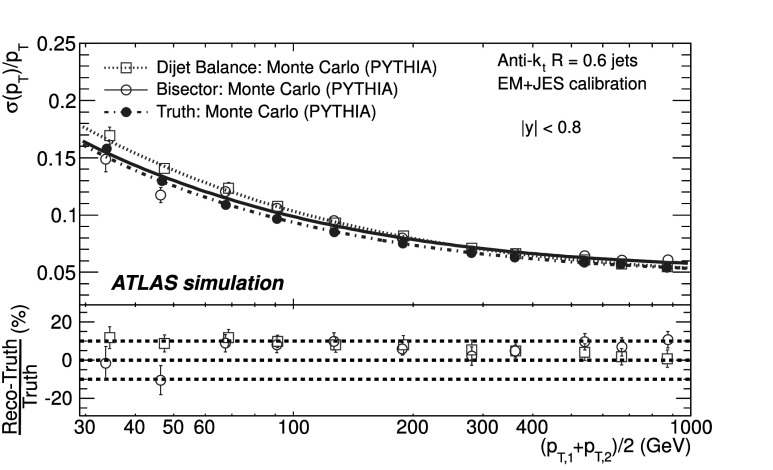
Comparison between the Monte Carlo simulation truth jet *p*_T_ resolution and the results obtained from the bisector and dijet balance in situ methods (applied to Monte Carlo simulation) for the EM+JES calibration, as a function of $\bar{p}_{\mathrm{T}}$. The curves correspond to fits with the functional form in Eq. (9). The *lower panel* of the figure shows the relative difference between the in situ methods and the fit to the Monte Carlo truth results. The *dotted lines* indicate a relative difference of ±10 %. The errors shown are only statistical

The Monte Carlo simulation truth jet *p*_T_ resolution is compared to the results obtained from the dijet balance and the bisector in situ methods (applied to Monte Carlo simulation) in Fig. 10. This comparison will be referred to as the closure test. The in situ and truth resolutions agree within 10 %, with the truth results typically 10 % lower. This result confirms the validity of the physical assumptions discussed in Sects. 6 and 7 and the inference that the observables derived for the in situ MC dijet balance and bisector methods provide reliable estimates of the jet energy resolution. The systematic uncertainties on these estimates are of the order of 10 % (15 %) for jets with *R*=0.6 (*R*=0.4), and are discussed in Sect. 10.

## Jet energy resolution uncertainties

There are three kind of systematic uncertainties to be considered. Section 10.1 discusses the experimental uncertainties that affect the in situ measurements. Section 10.2 addresses the method uncertainties, that is the precision with which the in situ methods in data describe the truth resolution. Finally, Sect. 10.3 studies the truth resolution uncertainty due to event modelling in the Monte Carlo simulation.

### Experimental in situ uncertainties

**Fig. 11:**
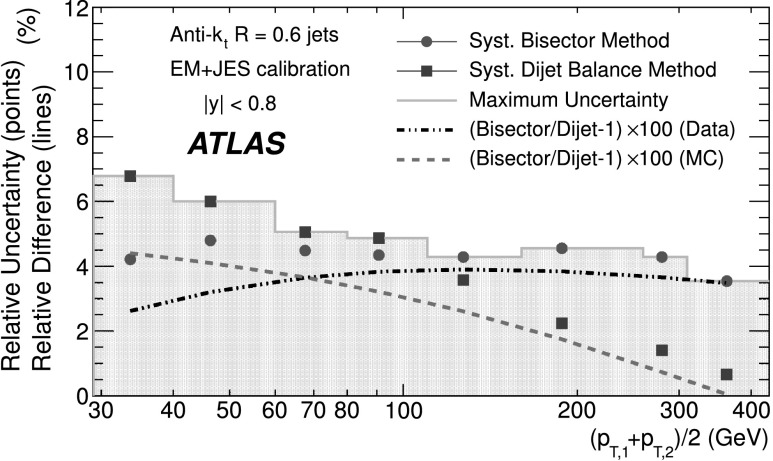
The experimental systematic uncertainty on the dijet balance (*squares*) and bisector (*circles*) methods as a function of $\bar {p}_{\mathrm{T}} $, for jets with |*y*|<0.8. Also shown is the absolute value of the relative difference between the two methods in each *p*_T_ bin for data (*dot-dashed lines*) and for Monte Carlo simulation (*dashed lines*)

The squares (circles) in Fig. 11 show the experimental relative systematic uncertainty in the dijet balance (bisector) method as a function of $\bar {p}_{\mathrm{T}}$. The different contributions are discussed below. The shaded area corresponds to the larger of the two systematic uncertainties for each $\bar{p}_{\mathrm{T}}$ bin.

For the dijet balance method, systematic uncertainties take into account the variation in resolution when applying different Δ*ϕ* cuts (varied from 2.6 to 3.0), resulting in a 2–3 % effect for 30≤*p*_T_<60 GeV, and when varying the parameterization of $K_{\mathrm{soft}}(\bar{p}_{\mathrm{T}})$ (see Sect. 6.2), which contributes up to 6 % at *p*_T_≈ 30 GeV. For the bisector method, the relative systematic uncertainty is about 4–5 %, and is derived from the precision with which it can be verified that $\sigma^{2\ {\mathrm{calo}}}_{\psi}-\sigma^{2\ {\mathrm{calo}} }_{\eta}$ stays constant when varying the $p^{\text{EM-scale}}_{{\mathrm{T}},3}$ cut.

The contribution from the JES uncertainties [39] is common to both methods. It is 1–2 %, determined by re-calculating the jet resolutions after varying the JES within its uncertainty in a fully correlated way. The resolution has also been studied in simulated events with added pile-up events (i.e. additional interactions as explained in Sect. 3.3), as compared to events with one hard interaction only. The sensitivity of the resolution to pile-up is found to be less than 1 % for an average number of vertices per event of 1.9.

In summary, the overall relative uncertainty from the in situ methods decreases from about 7 % at *p*_T_=30 GeV down to 4 % at *p*_T_=500 GeV. Figure 11 also shows the absolute value of the relative difference between the two in situ methods, for both data and Monte Carlo simulation. They are found to be in agreement within 4 % up to 500 GeV, and consistent with these systematic uncertainties.

### Uncertainties on the measured resolutions

The uncertainties in the measured resolutions are dominated by the systematic uncertainties, which are shown in Table 1 as a percentage of the resolution for the four rapidity regions and the two jet sizes considered, and for characteristic ranges, low (∼50 GeV), medium (∼150 GeV) and high (∼400 GeV) *p*_T_. The results are similar for the four calibration schemes.

**Table 1:** Relative systematic uncertainties on the measured resolutions at low (∼50 GeV), medium (∼150 GeV) and high (∼400 GeV) *p*_T_, for the four rapidity regions and the two jet radii studied. The uncertainties are similar for the four calibration schemes, and are dominated by the contributions from closure and data/MC agreement

Jet radius	Rapidity range	Total systematic uncertainty		
		Low *p*_T_	Med *p*_T_	High *p*_T_
*R*=0.6	0.0≤|*y*|<0.8	12 %	10 %	11 %
	0.8≤|*y*|<1.2	12 %	10 %	13 %
	1.2≤|*y*|<2.1	14 %	12 %	14 %
	2.1≤|*y*|<2.8	15 %	13 %	18 %
*R*=0.4	0.0≤|*y*|<0.8	17 %	15 %	11 %
	0.8≤|*y*|<1.2	20 %	18 %	14 %
	1.2≤|*y*|<2.1	20 %	18 %	14 %
	2.1≤|*y*|<2.8	20 %	18 %	18 %

The dominant sources of systematic uncertainty are the closure and the data/MC agreement. The experimental systematic uncertainties, discussed in Sect. 10.1, are significantly smaller. The closure uncertainty (see Sect. 9), defined as the precision with which in simulation the resolution determined using the in situ method reproduces the truth jet resolution, is larger for *R*=0.4 than for *R*=0.6, smaller at high *p*_T_ than at low *p*_T_, and basically independent of the rapidity. The data/MC agreement uncertainty, the precision with which the MC simulation describes the data, is independent of *R*, larger at low and high *p*_T_ than at medium *p*_T_, and it grows with rapidity because of the increasingly limited statistical accuracy with which checks can be performed to assess it.

The systematic uncertainties in Table 1 for jets with *R*=0.4 are dominated by the contribution from the closure test. They decrease with increasing *p*_T_ and are constant for the highest three rapidity bins. The systematic uncertainties for jets with *R*=0.6 are consistently smaller than for the *R*=0.4 case, and receive comparable contributions from closure and data/MC agreement. They tend to increase with rapidity and are slightly lower in the medium *p*_T_ range. The uncertainty increases at high *p*_T_ for the end-cap, 2.1≤|*y*|<2.8, because of the limited number of events in this region.

### Uncertainties due to the event modelling in the Monte Carlo generators

**Fig. 12:**
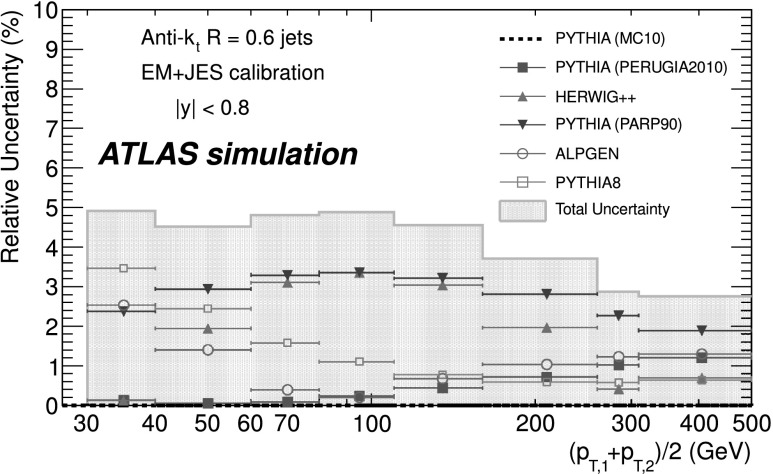
Systematic uncertainty due to event modelling in Monte Carlo generators on the expected jet energy resolution as a function of *p*_T_, for jets with |*y*|<0.8. The *points* correspond to absolute differences with respect to the results obtained with the nominal simulation (Pythia MC10). Other event generators are shown as *solid triangles* (Herwig++) and *open circles* (Alpgen). *Solid squares* (Pythia Perugia2010), *inverted triangles* (Pythia PARP90), and *open squares* (Pythia8), summarize differences coming from different tunes, cut-off parameters, and program version, respectively. The total modelling uncertainty is estimated from the sum in quadrature of the different cases considered here (*shaded area*)

Although not relevant for the in situ measurements of the jet energy resolution, physics analyses sensitive to the expected resolution have to consider its systematic uncertainty arising from the simulation of the event. The expected jet *p*_T_ resolution is calculated for several Monte Carlo simulations in order to assess its dependence on different generator models (Alpgen and Herwig++), Pythia tunes (Perugia2010), and other systematic variations (PARP90; see Sect. 3.1). Differences between the nominal Monte Carlo simulation and Pythia8 [24] have also been considered. These effects, displayed in Fig. 12, never exceed 4 %. The total modelling uncertainty is estimated from the sum in quadrature of the different cases considered here. This is shown by the shaded area in Fig. 12 and found to be at most 5 %.

## Jet energy resolution for other calibration schemes

**Fig. 13:**
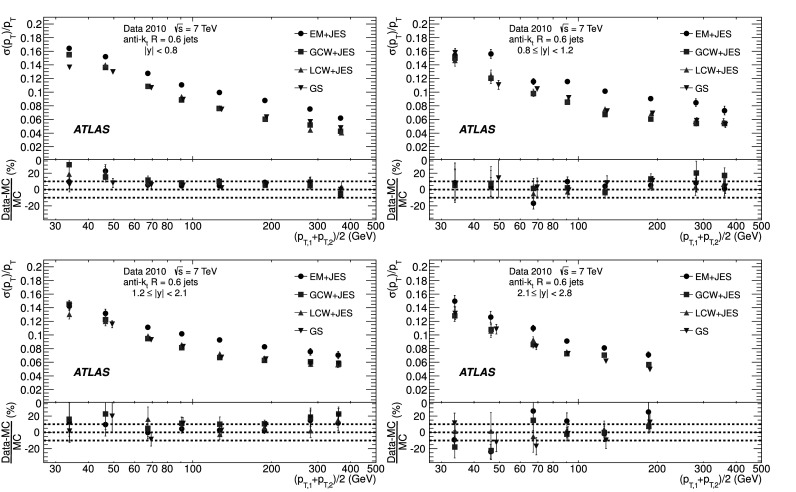
Fractional jet *p*_T_ resolutions as a function of $\bar{p}_{\mathrm{T}}$ for anti-*k*_*t*_ jets with *R*=0.6 with |*y*|<0.8 (*top left*), 0.8≤|*y*|<1.2 (*top right*), 1.2≤|*y*|<2.1 (*bottom left*) and 2.1≤|*y*|<2.8 (*bottom right*), using the bisector in situ method, for four jet calibration schemes: EM+JES, Local Cluster Weighting (LCW+JES), Global Cell Weighting (GCW+JES) and Global Sequential (GS). The *lower panels* show the relative difference between data and Monte Carlo simulation results. The *dotted lines* indicate relative differences of ±10 %. The errors shown are only statistical

The resolution performance for anti-*k*_*t*_ jets with *R*=0.6 reconstructed from calorimeter topological clusters for the Local Cluster Weighting (LCW+JES), the Global Cell Weighting (GCW+JES) and the Global Sequential (GS) calibration strategies (using the bisector method) is presented in Fig. 13 for the Central, Extended Tile Barrel, Transition and End-Cap regions. The top panel shows the resolutions determined from data, whereas the bottom part compares data and Monte Carlo simulation results. The three more sophisticated calibration techniques improve the resolution *σ*(*p*_T_)/*p*_T_ with respect to the EM+JES calibrated jets by approximately 0.02 over the whole *p*_T_ range. The relative improvement ranges from 10 % at low *p*_T_ up to 40 % at high *p*_T_ for all four rapidity regions.

**Fig. 14:**
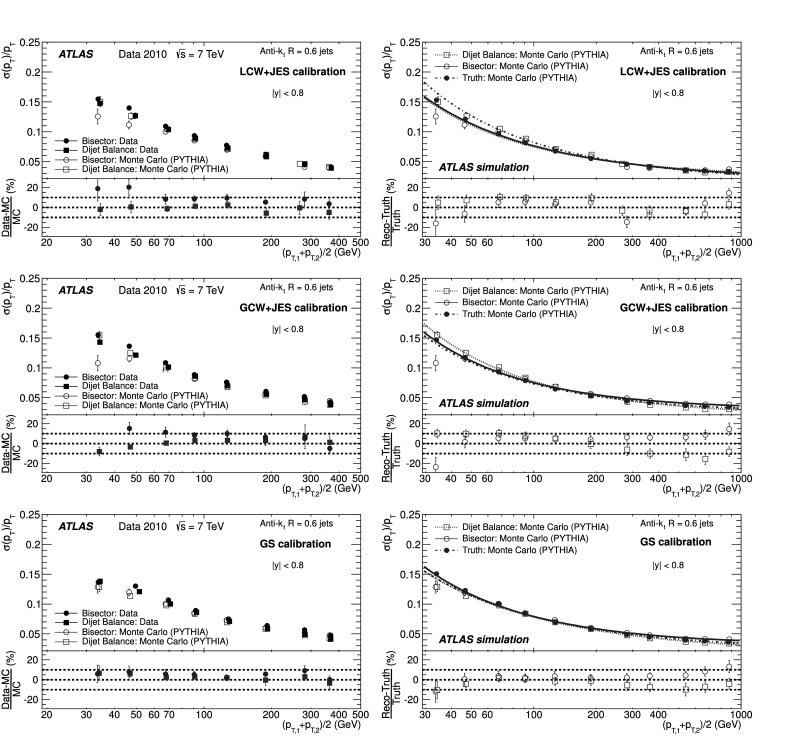
Fractional jet *p*_T_ resolutions as a function of $\bar {p}_{\mathrm{T}}$ for anti-*k*_*t*_ jets with *R*=0.6 for the Local Cluster Weighting (LCW+JES), Global Cell Weighting (GCW+JES) and Global Sequential (GS) calibrations. *Left*: Comparison of both in situ methods on data and MC simulation for |*y*|<0.8. The *lower panels* show the relative difference. *Right*: Comparison between the Monte Carlo simulation truth jet *p*_T_ resolution and the final results obtained from the bisector and dijet balance in situ methods (applied to Monte Carlo simulation). The *curves* correspond to fits with the functional form in Eq. (9). The *lower panel* of the figure shows the relative difference between the in situ methods and the fit to the Monte Carlo truth results. The *dotted lines* indicate relative differences of ±10 %. The errors shown are only statistical

Figure 14 displays the resolutions for the two in situ methods applied to data and Monte Carlo simulation for |*y*|<0.8 (left plots). It can be observed that the results from the two methods agree, within uncertainties. The Monte Carlo simulation reproduces the data within 10 %. The figures on the right show the results of a study of the closure for each case, where the truth resolution is compared to that obtained from the in situ methods applied to Monte Carlo simulation data. The agreement is within 10 %. Overall, comparable agreement in resolution is observed in data and Monte Carlo simulation for the EM+JES, LCW+JES, GCW+JES and GS calibration schemes, with similar systematic uncertainties in the resolutions determined using in situ methods.

## Improvement in jet energy resolution using tracks

The addition of tracking information to the calorimeter-based energy measurement is expected to compensate for the jet-by-jet fluctuations and improve the jet energy resolution (see Sect. 5.5).

**Fig. 15:**
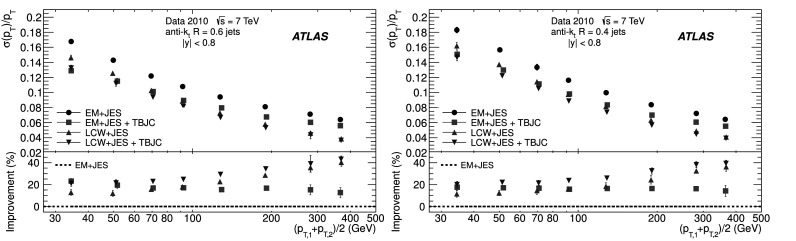
*Top*: Fractional jet *p*_T_ resolutions as a function $\bar {p}_{\mathrm{T}} $, measured in data for anti-*k*_*t*_ jets with *R*=0.6 (*top*) and *R*=0.4 (*bottom*) and for four jet calibration schemes: EM+JES, EM+JES+TBJC, LCW+JES and LCW+JES+TBJC. The *lower panel* of the figure shows the relative improvement for the EM+JES+TBJC, LCW+JES and LCW+JES+TBJC calibrations with respect to the EM+JES jet calibration scheme, used as reference (*dotted line*). The errors shown are only statistical

The performance of the Track-Based Jet Correction method (TBJC) is studied by applying it to both the EM+JES and LCW+JES calibration schemes, in the central region. The measured resolution for anti-*k*_*t*_ jets with *R*=0.6 (*R*=0.4) is presented as a function of the average jet transverse momentum in the top (bottom) plot of Fig. 15.

The relative improvement in resolution due to the addition of tracking information is larger at low *p*_T_ and more important for the EM+JES calibration scheme. It ranges from 22 % (10 %) at low *p*_T_ to 15 % (5 %) at high *p*_T_ for the EM+JES (LCW+JES) calibration. For *p*_T_<70 GeV, jets calibrated with the EM+JES+TBJC scheme show a similar performance to those calibrated with the LCW+JES+TBJC scheme. Overall, jets with LCW+JES+TBJC show the best fractional energy resolution over the full *p*_T_ range.

## Summary

The jet energy resolution for various JES calibration schemes has been measured using two in situ methods with a data sample corresponding to an integrated luminosity of 35 pb^−1^ collected in 2010 by the ATLAS experiment at $\sqrt{s}= 7\mbox{ TeV}$.

The Monte Carlo simulation describes the jet energy resolution measured in data within 10 % for jets with *p*_T_ values between 30 GeV and 500 GeV in the rapidity range |*y*|<2.8.

The resolutions obtained applying the in situ techniques to Monte Carlo simulation are in agreement within 10 % with the resolutions determined by comparing jets at calorimeter and particle level. Overall, the results measured with the two in situ methods have been found to be consistent within systematic uncertainties.

## Footnotes

1 The ATLAS reference system is a Cartesian right-handed coordinate system, with the nominal collision point at the origin. The anti-clockwise beam direction defines the positive *z*-axis, with the *x*-axis pointing to the centre of the LHC ring. The angle *ϕ* defines the direction in the plane transverse to the beam (*x*,*y*). The pseudorapidity is given by $\eta= -\ln\tan\frac{\theta}{2}$, where the polar angle *θ* is taken with respect to the positive *z* direction. The rapidity is defined as *y*=0.5×ln[(*E*+*p*_*z*_)/(*E*−*p*_*z*_)], where *E* denotes the energy and *p*_*z*_ is the component of the momentum along the *z*-axis.
